# A Design-Based VR-Assisted Learning System for Culturally Grounded Meaning Construction in Foreign Language Learning: Integrating Chinese Excellent Traditional Culture

**DOI:** 10.3390/bs16101718

**Published:** 2026-09-22

**Authors:** Liyuan Zeng, Jin Cui, Xinqi Su, Xiaomeng Jiang, Luxi Yang, Yishi Huang, Zhi Lu, Ning Zhang, Yongqiang Jiang, Ruixian Li, Xiaojin Liu

**Affiliations:** 1 Faculty of Arts and Sciences, Beijing Normal University, Zhuhai 519087, China; 2 Faculty of Education, Beijing Normal University, Beijing 100875, China; 3 College of Foreign Languages, Huaqiao University, Quanzhou 362021, China; 4 Center for Educational Science and Technology, Beijing Normal University, Zhuhai 519087, China; 5 College of Education for the Future, Beijing Normal University, Zhuhai 519087, China

**Keywords:** virtual reality, language teaching, Chinese Excellent Traditional Culture, immersive learning, virtual reality-assisted language learning

## Abstract

This study investigates how a VR-assisted language learning system can be designed to support culturally grounded meaning construction in foreign language learning through the integration of Chinese Excellent Traditional Culture (CETC). Drawing on cultural linguistics and cognitive–affective perspectives, our study adopted a multi-phase design-based research approach consisting of three interconnected studies. Study 1 employed a questionnaire survey to examine learners’ challenges and needs in expressing CETC in foreign languages. The findings revealed three major challenges: insufficient contextualized cultural input, difficulties in mapping cultural meanings to linguistic forms, and affective barriers such as anxiety and low communicative confidence. Based on these findings, Study 2 developed a VR-assisted language learning system integrating immersive cultural scenarios, multimodal input, and interactive communicative tasks. Study 3 further explored learners’ perceptions and experiences through immersive VR sessions and semi-structured interviews. The findings suggest that learners perceived VR as potentially supporting contextualized cultural understanding, facilitating the perceived alignment between cultural meanings and language expression, and creating a relatively low-anxiety space for communication practice. The study suggests that the potential educational value of VR may lie not in immersion itself, but in its perceived capacity to mediate the interaction between cultural knowledge, language use, and affective engagement in culturally situated learning environments.

## 1. Introduction

In the context of globalization, the shift in foreign language education (FLE) from a sole focus on linguistic competence, such as grammar and vocabulary, to the integration of intercultural communicative competence (ICC) has been advocated for a considerable time (Avgousti, 2018; Byram, 2020; Iswandari & Ardi, 2022; Zhou et al., 2024). Within this paradigm shift, Byram’s ICC model provides a widely adopted theoretical framework, emphasizing five core dimensions: attitudes toward cultural difference, knowledge of target and native cultures, skills of interpreting cultural meanings, skills of intercultural interaction, and critical cultural awareness, highlighting that language learning is inherently intertwined with cultural understanding (Byram, 2020). Most curricular frameworks, including the Common European Framework of Reference for Languages (CEFR), have further foregrounded cultural competence as a key component of language education (Council of Europe, 2001). Recent studies have continued to expand this perspective, highlighting the need for learners not only to understand target-language cultures but also to engage reflexively with their own cultural identities in intercultural encounters (Z. Qu & Chen, 2026; Wibowo et al., 2024). These reflect the growing recognition that language functions not only as a communicative system but also as a medium of cultural meaning, encompassing both linguistic forms and their cultural connotations. Nevertheless, questions remain regarding how cultural knowledge can be coherently integrated into technology-mediated language learning environments, where design decisions shape not only linguistic input but also cultural representation.

Despite the global emphasis on cultural integration, Chinese students frequently encounter the challenge of “cultural aphasia”, a phenomenon where learners can communicate fluently about Western cultural topics (e.g., Christmas traditions, Shakespearean plays) in a foreign language but struggle to express Chinese Excellent Traditional Culture (CETC) effectively (Peng, 2017; J. Song & Bai, 2018; M. Yan, 2022). In this study, CETC refers to selected elements of Chinese traditional culture that are culturally representative, educationally valuable, and suitable for integration into foreign language learning, including traditional festivals, rituals, values, arts, symbols, and everyday cultural practices. This challenge reflects a structural imbalance in intercultural competence development, whereby target-language cultural content is extensively embedded in instructional materials and communicative practices, while learners’ native cultural resources remain comparatively underrepresented (Kim, 2014; Tok & Tavil, 2024). As a result, learners may develop familiarity with foreign cultural references yet lack the linguistic and discursive means to express culturally situated knowledge rooted in their own traditions. This imbalance is further reinforced by the limited integration of Chinese cultural scenarios into mainstream English language teaching materials and classroom activities, which often prioritise Western sociocultural contexts. Consequently, the challenge lies not only in acquiring cultural knowledge, but in designing learning environments that enable learners to meaningfully mobilise their own cultural identities in foreign language communication.

In light of the challenges associated with integrating learners’ native cultural resources into foreign language education, virtual reality (VR) has been increasingly recognised as a promising medium for culturally situated language learning (Parmaxi, 2023; Tafazoli, 2024). From a cognitive perspective, VR may support cognitive processing by embedding linguistic input within immersive and contextually meaningful environments, thereby facilitating comprehension and knowledge integration (Chen & Kent, 2020; W. Liu, 2025). From an affective perspective, VR environments have been reported to potentially enhance learner engagement, intrinsic motivation, and self-efficacy by providing interactive and low-anxiety spaces for communication (Qiu et al., 2024; Sun & Song, 2025). These affordances suggest that VR has the potential to support not only language acquisition but also culturally grounded meaning-making, making it particularly suitable for addressing imbalances in cultural representation within foreign language learning.

However, despite these theoretical advantages, existing VR-based language learning systems have yet to fully realise this potential. Current applications tend to prioritise scenarios grounded in Anglo-American or European sociocultural contexts, implicitly positioning them as default environments for language use. Such tendencies constrain learners’ opportunities to engage with culturally diverse or locally relevant content, thereby reproducing the imbalance identified in traditional foreign language instruction. Moreover, previous studies have noted that VR-based learning environments often emphasise technological immersion over pedagogical and cultural alignment, resulting in experiences that are engaging but not necessarily culturally meaningful or instructionally effective (Bekele & Champion, 2019; Rudling et al., 2026). In the Chinese context, VR language learning applications remain at an early stage of development and rarely incorporate culturally authentic representations of Chinese traditional practices. Consequently, there is a pressing need to develop VR-based language learning systems that integrate local cultural resources in a theoretically grounded and pedagogically coherent manner.

The present study therefore explores how a culturally grounded and pedagogically coherent VR-assisted language learning prototype can be conceptualized, designed, and perceived in the context of Chinese higher education. Rather than testing the learning effectiveness of VR, the study focuses on learners’ needs, prototype development, and exploratory learner-perception evidence. To this end, the study is guided by the following research questions:

**RQ1.**  *What challenges do learners face in constructing culturally grounded meanings in language use?*

**RQ2.**  *How can a CETC-integrated VR-assisted language learning system be designed to respond to these learner-reported challenges?*

**RQ3.**  *How do learners perceive and experience the role of VR in supporting culturally grounded meaning construction in language learning?*

## 2. Literature Review

### 2.1. Cultural Linguistics and Cultural Conceptual Transfer in Second Language Learning

Cultural linguistics conceptualises language as a system that both reflects and shapes culturally shared ways of thinking, thereby foregrounding the inseparability of language and culture in meaning-making (Sharifian, 2015, 2017; Zou et al., 2025). From this perspective, language learning extends beyond the acquisition of grammatical forms and lexical items to include engagement with culturally embedded conceptual structures, such as schemas, metaphors, and value systems. This view has important implications for second language learning (SLL), where successful communication depends not only on linguistic accuracy but also on the ability to appropriately interpret and convey culturally situated meanings.

A pivotal concept linking cultural linguistics and second language learning (SLA) is cultural conceptual transfer, defined as the process by which learners draw on culturally grounded cognitive frameworks from their first language (L1) when engaging with a second language (L2) (Jarvis, 2016; Sharifian, 2008). These frameworks encompass culturally specific schemas, implicit norms, and patterns of interpretation that guide the construction and expression of meaning. Previous empirical studies have primarily examined this process in relation to learners’ pragmatic failure, metaphor interpretation, and intercultural miscommunication in L2 use, consistently showing that learners tend to rely on L1-based conceptual systems when interpreting culturally loaded expressions, often leading to mismatches in meaning construction (Altakhaineh et al., 2024; Ifantidou, 2017; J. Qu & Miwa, 2025). While such a transfer may facilitate communication when conceptual systems are aligned, it can also impede effective expression when culturally embedded meanings are not adequately adapted to the target language context (Melinte, 2012). Accumulating evidence further indicates that even advanced L2 learners experience persistent difficulty in expressing culturally specific meanings, not due to linguistic deficiency but due to challenges in reconciling divergent conceptual systems. For example, J. Qu and Miwa (2025) show that Japanese learners of English exhibit only partial shifts toward target-language event construal patterns, continuing to rely on L1-based conceptual biases, while Mjilla and Darir (2025) demonstrate that idioms lacking shared conceptual metaphors across languages pose significant challenges for bilingual learners. Taken together, these findings suggest that difficulties in L2 use are deeply rooted in mismatches between culturally shaped conceptual systems, constraining both the comprehension and expression of culturally specific meanings. In this sense, the phenomenon of “cultural aphasia” observed among Chinese learners of foreign languages can be understood as a failure to regulate cultural conceptual transfer, whereby learners possess sufficient linguistic resources yet struggle to articulate culturally grounded concepts due to difficulties in recontextualising culturally embedded meanings.

This challenge is especially pertinent in the context of CETC, which is characterised by its rich connotations, implicit value systems, and context-dependent meanings (S. Liu, 2023; L. Song, 2021; Y. Wang, 2025). In the absence of adequate pedagogical support, learners often resort to literal translation or simplified explanations, resulting in partial or distorted representations of cultural meanings (Gebbia, 2023; Q. Wang et al., 2025). Melinte (2012) notes that syntactic-level cultural transfer is often overlooked in traditional FLE, which focuses on decontextualized grammar teaching and fails to connect syntactic structures to cultural cognitive differences. This gap is a key reason for the “surface-level cultural competence” of Chinese learners. These limitations point to a broader issue in traditional foreign language education, which has struggled to effectively support learners in regulating cultural conceptual transfer (Huang, 2023).

### 2.2. Cognitive and Affective Mechanisms of Immersive VR-Assisted Language Learning

VR has been increasingly examined as a medium for language learning because it can combine immersive experience, interaction, and contextualized language input (Sun & Song, 2025; Vallarino et al., 2025; Zappa et al., 2025). Previous reviews and empirical studies suggest that VR may support vocabulary learning, speaking practice, and intercultural learning, particularly when immersive features are pedagogically aligned with meaningful tasks (W. Liu, 2025; Qiu et al., 2024; Sun & Song, 2025). However, these potential benefits depend not on immersion alone, but on how VR environments are designed to connect language input, learner interaction, and task engagement (Klingenberg et al., 2024; Makransky & Petersen, 2021; Shin, 2017). Immersive VR should therefore not be assumed to automatically reduce cognitive load. While contextualized visual cues may support comprehension, learners may also experience increased attentional and operational demands when they need to process linguistic input, visual information, spatial navigation, controller operation, and task instructions simultaneously. Thus, VR-assisted language learning requires careful instructional and interface design to ensure that immersive features support rather than compete with language processing.

The Cognitive Affective Model of Immersive Learning (CAMIL) provides an integrative theoretical framework for understanding how immersive features may translate into learning outcomes (Makransky & Petersen, 2021). Rather than proposing entirely new learning principles, CAMIL synthesises existing evidence on immersive learning by specifying how technological features of VR give rise to learning-relevant psychological processes. Within this framework, the educational impact of VR is understood as being mediated by key psychological affordances, most notably presence and agency, which arise from immersive environments. Presence refers to the learner’s subjective sense of “being there”, while agency captures the extent to which learners can actively control and interact with the virtual environment. In VR-assisted language learning, these affordances are not merely experiential; rather, they shape how learners allocate attention, process linguistic input, and engage in language tasks (Chen & Kent, 2020; Hiver et al., 2025). For example, heightened presence has been associated with increased attentional engagement and sustained involvement in communicative activities, while greater agency enables learners to participate more actively in interaction, such as navigating scenarios, manipulating objects, and responding to virtual interlocutors in real time (Chen & Kent, 2020; Zappa et al., 2025). These attentional and interactional processes are particularly relevant for task-based and scenario-based language learning, where successful performance depends on the integration of linguistic, contextual, and interactional resources (Hiver et al., 2025). Although originally developed as a general model of immersive learning, CAMIL has been increasingly applied in VR-assisted language learning research to explain how learners engage in communicative tasks and how such engagement may contribute to language learning outcomes, including vocabulary acquisition, speaking performance, and knowledge transfer.

### 2.3. Situated and Embodied Mechanisms of Culturally Grounded Meaning Construction in VR-Assisted Language Learning

According to situated learning theory, knowledge is constructed through participation in contextually meaningful activities rather than through decontextualized instruction (Bloch, 1994; Durning & Artino, 2011). In VR-based language learning, this principle is operationalized through the creation of immersive sociocultural environments in which language is embedded in authentic or semi-authentic contexts (Li & Jeong, 2020; Zappa et al., 2025). Previous studies have shown that such environments enable learners to engage in contextually grounded communication, where linguistic forms are closely tied to social roles, norms, and interactional expectations (Li & Jeong, 2020; Rudling et al., 2026; Zappa et al., 2025). Rather than passively receiving input, learners participate in culturally situated practices, such as role-playing everyday interactions or completing scenario-based tasks, which have been found to support the development of pragmatic competence and context-sensitive language use (J. Liu, 2025; Rudling et al., 2026; Yeh et al., 2022). In this sense, VR facilitates a shift from decontextualized language learning toward forms of engagement in which meaning is constructed through the integration of linguistic and sociocultural information.

In addition to contextualization, embodied interaction constitutes a key mechanism through which VR supports culturally grounded meaning construction (Radianti et al., 2020). Based on embodied cognition theory, cognitive processing is shaped by sensorimotor and experiential engagement (Davis & Markman, 2012; Kiefer & Trumpp, 2012). In VR environments, learners can interact with virtual objects, navigate social spaces, and respond to multimodal cues, thereby linking linguistic expressions to concrete experiential and cultural referents (Pan et al., 2025; Senkbeil, 2025). Empirical evidence indicates that such embodied engagement can enhance learners’ understanding of culturally embedded meanings, particularly in tasks involving object manipulation, spatial interaction, or socially situated communication (Kenderdine, 2015; Lee et al., 2024; Zappa et al., 2025). Moreover, previous studies have shown that participation in immersive cultural scenarios, such as virtual cultural events or simulations of everyday social practices, can help learners connect language use with underlying cultural values, norms, and interactional scripts, rather than treating language as an abstract system (Pan et al., 2025; Rudling et al., 2026; Yeh et al., 2022). These findings suggest that VR supports an integrated process of meaning construction in which language use is grounded in culturally situated experience. However, despite these advances, existing research has largely focused on general cultural exposure or task-based performance, with limited attention to how specific cultural conceptual systems are represented and internalized in VR-based language learning contexts, particularly in relation to learners’ ability to construct culturally appropriate meanings in use.

### 2.4. Research Gap and Current Study

Despite growing interest in VR-assisted language learning, limited attention has been paid to how VR can be designed to help learners express their own cultural knowledge in a foreign language. Existing studies and applications have tended to emphasize functional communication, vocabulary learning, target-culture immersion, or surface-level cultural representation, while the integration of local cultural resources such as CETC and the alignment between cultural meanings and linguistic forms remain underexplored. To address these gaps, this study first investigates learners’ perceived challenges in expressing CETC in foreign languages, then develops a CETC-integrated VR prototype informed by cultural linguistics and cognitive-affective learning principles, and finally explores learners’ perceptions and experiences after a short VR interaction.

## 3. Methods

### 3.1. Overall Research Design

This study adopted a multi-phase design-based research (DBR) approach to investigate and support culturally grounded meaning construction in language learning through VR. The research comprised three interconnected phases aligned with the research questions. In Study 1, a large-scale questionnaire survey was conducted to identify learners’ challenges and needs in constructing culturally grounded meanings in language use, serving as a needs analysis stage. In Study 2, a VR-based language learning system was designed and developed based on cultural linguistics and cognitive–affective perspectives, translating theoretical principles and learner needs into concrete design features and system architecture. In Study 3, an exploratory qualitative study involving immersive VR experiences and semi-structured interviews was conducted to examine learners’ perceptions and experiences of the system. Together, the three phases followed a coherent logic of needs analysis, theory-informed design, and exploratory user-experience evaluation, with quantitative and qualitative data used to provide a comprehensive understanding of learners’ needs, the design rationale of the VR system, and learners’ perceived experiences of the proposed VR-based approach. The protocol was approved by the Institutional Review Board (IRB) of Faculty of Education, Beijing Normal University. Written informed consent was obtained from all subjects prior to the study. All procedures performed were following the ethical standards of the appropriate institutional research boards and with the 1964 Helsinki declaration and its later amendments or comparable ethical standards.

### 3.2. Study 1: Challenges in Culturally Grounded Meaning Construction

#### 3.2.1. Participants

A total of 292 responses were initially collected through the online questionnaire survey. After data screening, 15 responses were excluded, and the final valid sample consisted of 277 college students enrolled in universities across China. Among them, 206 were female students (74.37%) and 71 were male students (25.63%). In terms of academic level, the majority were freshmen (*N* = 85, 30.69%) and sophomores (*N* = 134, 48.38%), followed by juniors (*N* = 32, 11.55%), seniors (*N* = 12, 4.33%), postgraduate students (*N* = 11, 3.97%), doctoral students (*N* = 1, 0.36%), and other participants (*N* = 2, 0.72%).

Participants represented diverse disciplinary backgrounds. This item was set as a multiple-response question, and participants were allowed to select more than one discipline. Therefore, the following figures refer to selection frequencies, with percentages calculated based on the total sample size (*N* = 277): Science, Technology, Engineering, and Mathematics (*N* = 116, 41.88%), Humanities and Social Sciences (*N* = 74, 26.71%), language-related majors (*N* = 66, 23.83%), Arts and Physical Education (*N* = 10, 3.61%), Economics and Management (*N* = 9, 3.25%), and other fields (*N* = 10, 3.61%). Regarding prior experience with VR, 126 participants (45.49%) reported having used VR technology, whereas 151 participants (54.51%) had no prior exposure.

#### 3.2.2. Instruments

A self-developed needs-analysis questionnaire was designed to examine learners’ perceived challenges, needs, and expectations regarding the integration of CETC into foreign language learning and the potential use of VR-supported learning environments (VR-Supported Foreign Language Learning Needs-Analysis Questionnaire). The questionnaire was not intended to function as a standardized psychometric scale or to measure latent constructs for hypothesis testing. Rather, it was developed as a descriptive needs-assessment instrument to inform the subsequent design and development of the VR-based learning system.

The final instrument consisted of 41 items, including demographic questions, single-choice items, multiple-response items, 24 five-point Likert-type items, and one open-ended question. The Likert-type items were scored from 1 = strongly disagree to 5 = strongly agree, with higher scores indicating stronger agreement with the corresponding statement. Categorical, single-choice, and multiple-response items were analyzed using frequencies and percentages. The questionnaire covered four content domains: background information, perceptions and needs regarding CETC integration, prior VR usage and learning experience, and expectations for CETC-integrated VR-supported learning systems. These domains were used to organize the needs analysis and should not be interpreted as psychometrically validated subscales.

The questionnaire was developed based on the research objectives and relevant literature on intercultural language learning, culturally situated learning, and VR-assisted language learning. To improve content relevance and clarity, the initial version was reviewed by experts in foreign language education, educational technology, and intercultural communication. A pilot test was then conducted with 30 college students to assess item clarity and comprehensibility. Based on expert comments and feedback, ambiguous wording was revised before formal administration. The full needs-analysis questionnaire (both the original Chinese version and the English translation), including item wording, response formats, and scoring rules, is provided in File S1.

#### 3.2.3. Procedure

The study was conducted through an online survey administered over a three-week period. The research procedure involved three stages.

First, the questionnaire was developed based on the research objectives, relevant literature, and the design needs of the subsequent VR system. A pilot test was conducted with 30 college students to assess the clarity, readability, and comprehensibility of the items. Based on participant feedback, ambiguous or unclear items were revised.

Second, the finalized questionnaire was distributed via online platforms, including university student groups, social media channels, and academic networks. Prior to participation, respondents were informed of the study’s purpose, assured of confidentiality and anonymity, and notified that participation was voluntary.

Third, data screening was conducted to ensure response quality. A total of 292 responses were initially collected. Responses were excluded based on two operational criteria. Specifically, three responses were excluded because they contained clearly invalid age entries, including one-digit or three-digit values that were inconsistent with the university-student sample. The remaining 12 responses were excluded because they showed straight-lining response patterns, with the same option selected across all questionnaire items, and were completed in only slightly more than 10 s. After screening, 15 responses were excluded, and 277 valid responses were retained, yielding a valid response rate of 94.86%. All subsequent analyses in Study 1 were based on the final valid sample of 277 participants.

#### 3.2.4. Data Analysis

All data were analyzed using SPSS 26.0. Given the needs-analysis purpose of Study 1, the analysis was primarily descriptive. For Likert-type items, means, standard deviations, and the percentage of participants selecting “agree” or “strongly agree” were reported. The agreement percentage was used to identify salient learner needs and perceived challenges, while means and standard deviations were provided to improve the transparency and interpretability of the results. For categorical and multiple-response items, frequencies and percentages were calculated, with the valid denominator specified for each analysis. After data screening, no missing values were found for the retained Likert-type items because incomplete responses had been excluded before analysis.

No exploratory or confirmatory factor analysis was conducted in the revised analysis because the questionnaire was not designed as a standardized latent-variable scale. The Likert-type items represented heterogeneous content indicators related to learners’ needs, expectations, and perceptions, rather than reflective indicators of a single underlying construct. Therefore, item-level descriptive results were considered more appropriate for the purpose of informing the design of the VR-based learning system.

### 3.3. Study 2: Design and Development of the VR-Based Learning System

Study 2 focused on the design and development of a theory-informed VR-assisted language learning prototype. It was not intended to provide empirical evidence of learning effectiveness, but to translate the learner needs identified in Study 1 and the theoretical principles of cultural linguistics and cognitive–affective learning into concrete system features.

#### 3.3.1. Theoretical Foundations

The design of the VR-based language learning system was grounded in the integration of two complementary theoretical frameworks: cultural linguistics and multimedia affective–cognitive theory. Together, these frameworks informed the development of a system aimed at supporting culturally grounded meaning construction through the integration of CETC.

From the perspective of cultural linguistics, language is viewed as inherently embedded in cultural conceptual systems. Accordingly, three principles guided the design. First, the language–culture symbiosis principle emphasizes that linguistic expressions are inseparable from their underlying cultural meanings (e.g., tuanyuan [reunion] reflects the cultural value of family unity). Second, the cultural conceptual alignment principle requires that learning content authentically represents core CETC values (e.g., collectivism in festival practices and harmony with nature in traditional philosophy), thereby avoiding decontextualized cultural presentation. Third, the cultural conceptual transfer principle highlights the need to support learners in recognizing and regulating potential mismatches between native and target cultural conceptual systems.

Complementing this, multimedia affective–cognitive theory informed the system’s design in terms of cognitive processing and emotional engagement. The modality principle guided the integration of auditory (e.g., native-speaker pronunciation) and visual (e.g., immersive cultural scenes) channels to optimize cognitive load. The emotional design principle was applied to create culturally resonant scenarios (e.g., festive joy or ritual solemnity), thereby fostering positive affect and sustained engagement. Finally, the interaction principle emphasized active learner participation through embodied and task-based interactions, supporting deeper integration of linguistic and cultural processing.

#### 3.3.2. Design Principles

Based on the theoretical foundations and informed by the findings of Study 1, the system design followed four key principles.

First, the principle of culturally grounded meaning construction guided the overall design, ensuring that language learning activities were embedded in meaningful cultural contexts rather than presented as isolated linguistic forms.

Second, the principle of cultural specificity and authenticity emphasized the integration of CETC as core content. Cultural scenarios (e.g., traditional festivals, daily practices, and social interactions) were designed to reflect authentic cultural values, practices, and conceptual systems.

Third, the principle of cognitive-affective demand management aimed to balance cognitive, attentional, and affective demands during VR-based learning. Multimodal input, emotionally congruent scenarios, and step-by-step interactive tasks were designed to support comprehension and engagement while avoiding unnecessary visual, operational, or task-related burden.

Fourth, the principle of adaptive and user-centered design was incorporated in response to learners’ needs identified in Study 1 (e.g., demand for adjustable difficulty and usability concerns). The system therefore included differentiated learning modes (e.g., scaffolded vs. immersive modes) and intuitive interaction mechanisms to enhance accessibility and reduce barriers to use.

#### 3.3.3. System Architecture

The system was developed using a three-layer architecture that translates theoretical principles into functional components: the theoretical layer, the design layer, and the outcome layer (Figure 1).

The theoretical layer establishes the conceptual foundation of the system by integrating cultural linguistics and multimedia affective–cognitive theory, ensuring that all design decisions align with both cultural authenticity and cognitive–affective effectiveness.

The design layer operationalizes these principles through three core functional modules:

(1) Cultural Content Module:

This module provides culturally rich learning content centered on key domains of CETC, including major traditional festivals (e.g., Spring Festival, Qingming Festival, Dragon Boat Festival, and Mid-Autumn Festival) and representative cultural symbols (e.g., traditional architecture and arts). Interactive elements are designed to allow learners to explore cultural meanings embedded in these contexts, and are intended to support culturally grounded meaning construction.

(2) Language Skill Module:

This module integrates core language skills, including listening, reading, and speaking, within CETC contexts. It provides multimodal input (e.g., audio pronunciation and bilingual texts) and is designed to support productive skills through speech recording and feedback, intended to facilitate the integration of linguistic forms with culturally grounded meanings.

(3) Situational Immersion Module:

This module creates immersive and interactive VR environments through high-fidelity scene reconstruction, real-time feedback, and scenario-based communicative tasks. Learners are able to engage in culturally situated activities, which is intended to support experiential learning and contextualized meaning construction.

The system supports multiple target languages (English, French, and Japanese) and incorporates adaptive difficulty levels, including a scaffolded mode with native-language support and a more immersive mode using only the target language.

The outcome layer defines the intended learning outcomes of the system, including (a) enhanced ability to construct culturally grounded meanings in language use, (b) improved ability to express CETC concepts in a foreign language, and (c) increased learner engagement and motivation in culturally enriched learning environments.

To make the interaction design more concrete, a representative example is the Spring Festival scenario (Figure 2). Before entering the virtual scene, the learner first completes a pre-scene reading-related activity based on a brief introduction to the Spring Festival. This introductory activity provides basic cultural background information and target-language input related to the festival. The learner can listen to the pronunciation of the passage, complete oral reading or follow-along practice, and receive AI-based scoring as formative feedback. After entering the virtual scene, the learner encounters a virtual foreign peer and receives the prompt to introduce the Spring Festival in the target language. The learner then selects a cultural object, such as “dumpling”, after which the system presents two activity options: vocabulary learning and dialogue practice. In the vocabulary-learning activity, the learner can view the target word, listen to its pronunciation, and complete oral imitation with AI-based scoring. In the dialogue-practice activity, the virtual peer first produces a prompt utterance, and the learner selects one response from several candidate target-language replies. The learner may listen to each candidate response, but only one response is selected for oral practice and scoring. In the scaffolded mode, Chinese prompts are provided; in the immersive mode, only the target language is displayed. After the learner’s oral response is scored, the dialogue proceeds to the next utterance by the virtual peer. At the end of the activity, the system presents a brief performance summary, including vocabulary-learning and dialogue-practice scores, and the learner may either repeat the activity or proceed to the next cultural item.

The prototype included an AI-based oral-scoring function provided by the iFLYTEK Open Platform’s online speech assessment service (iFLYTEK Co., Ltd., Hefei, China). This function was used for fixed-reference reading assessment rather than open-ended speech recognition, dialogue generation, or content-understanding evaluation. After learners read a specified word, dialogue sentence, or festival-introduction sentence, the system recorded the voice, encoded the audio, and transmitted both the reference text and audio to the external service through an encrypted WebSocket connection. The returned scoring fields included overall score, pronunciation, fluency, integrity/completeness, and rhythm. The provider-returned overall score was displayed as the total score; the local system did not calculate a weighted composite score. The scores were mapped into four formative feedback levels: Excellent, Good, Pass, and Fail. These scores were used only as practice-oriented feedback and should not be interpreted as validated language-proficiency measures. The current system did not train a local scoring model, provide word-level or phoneme-level diagnostic feedback, or validate the scores against teacher ratings or standardized language tests. Raw audio files were not stored locally after scoring; the local learning records stored only information such as score, grade, practice text, language, difficulty level, and time. In terms of adaptivity, the current prototype provided predefined learner-selected modes rather than automatic performance-based adaptation. For example, learners could select an easier mode with Chinese prompts or a harder mode without Chinese prompts, but the system did not automatically adjust difficulty, content, or feedback based on learner performance.

#### 3.3.4. Development Process

The system was developed through an iterative design and implementation process, informed by both theoretical considerations and empirical findings from Study 1.

In terms of technical implementation, the system was developed using Unity 3D (version 2022.3.57f1c2; https://unity.com/cn/), Blender (version 4.2.2 LTS; https://www.blender.org/), and C# 9.0 to enable interactive and scalable VR environments. The hardware platform selected was Meta Quest 3 (Meta Platforms Technologies, LLC, Menlo Park, CA, USA), which allows for standalone operation and reduces the complexity of external device configuration. This choice was informed by usability concerns identified in Study 1.

Several optimizations were incorporated to enhance user experience. Local data storage was used to reduce latency, addressing a key issue reported by participants with prior VR experience. Additionally, motion sickness was mitigated through smooth navigation design and stable frame rates, following established VR usability guidelines.

The development process emphasized the alignment between user needs, theoretical principles, and technical feasibility. Specifically, findings from Study 1, such as learners’ demand for culturally rich content, adaptive difficulty, and ease of use, were directly translated into system features and interaction design.

Following system development, an exploratory user experience phase was conducted, in which participants engaged with the VR system and provided feedback. The design and findings of this evaluation are reported in Study 3, which focuses on learners’ perceptions and experiences of VR-supported culturally grounded meaning construction.

### 3.4. Study 3: Learners’ Perceptions and Experiences

#### 3.4.1. Participants

To explore learners’ perceptions and experiences of the VR-based language learning system, a total of 50 university students were recruited to participate in an exploratory VR experience session. This session was designed as a system trial rather than a formal instructional intervention. From this group, 10 participants were randomly selected for follow-up semi-structured interviews. The selected interviewees varied in academic background, language proficiency, and prior VR experience, enabling them to provide informed and diverse feedback on the system. These 10 interviewees constituted the qualitative interview sample for Study 3.

#### 3.4.2. Procedure

Study 3 was conducted after the completion of Study 1, the questionnaire survey, and Study 2, the system design and development, forming the final phase of the multi-stage research design.

The procedure consisted of three stages. First, all 50 participants participated in a standardized VR experience procedure that was designed to last approximately 30 min. According to the session records, 47 participants completed the full 30-min session, whereas three participants had shortened sessions lasting approximately 18, 20, and 25 min, respectively. During this session, they explored selected virtual scenarios integrating CETC with foreign language use. The session emphasized interaction and system exploration, allowing participants to become familiar with key features, including cultural scenarios, multimodal input, and task-based interaction. Second, immediately following the VR experience, 10 participants were randomly selected and invited to take part in individual semi-structured interviews. One of the selected interviewees was the participant who had completed approximately 20 min of the VR session and later reported dizziness and nausea during the interview. Third, interviews were conducted shortly after the VR session to ensure that participants’ reflections were grounded in their immediate experiences. This procedure enabled the collection of timely and contextually rich data regarding learners’ perceptions of the system and its perceived role in supporting culturally grounded meaning construction. Accordingly, Study 3 did not aim to measure learning gains or instructional effectiveness, but to explore learners’ immediate perceptions, experiences, and suggestions after interacting with the prototype system.

#### 3.4.3. Interview Design

Data were collected using a semi-structured interview protocol developed specifically for this study. The interview guide was informed by the findings of Study 1 and aligned with the design objectives of the VR system, ensuring coherence across research phases. The 19 interview questions were organized into four predefined modules. Questions 1–2 focused on learners’ prior exposure to CETC in foreign language learning; Questions 3–5 examined cultural confidence and foreign-language expression; Questions 6–9 addressed cross-cultural communication barriers and training needs; and Questions 10–19 explored learners’ VR learning experience and system refinement needs. This structure ensured that the interview protocol was aligned with the purposes of Study 3, namely, to contextualize the questionnaire findings, examine learners’ perceptions of the VR system, and identify directions for future system improvement. Each interview lasted approximately 20–30 min and was conducted individually in a quiet environment. With informed consent, all interviews were audio-recorded for accuracy and completeness. The complete semi-structured interview protocol (both the original Chinese version and the English translation) is provided in File S1.

#### 3.4.4. Data Analysis

Given the exploratory and formative purpose of Study 3, the qualitative analysis was conducted only on the interview transcripts from the 10 participants who completed the follow-up interviews. The interview data were analyzed using a framework-guided descriptive qualitative approach. The aim was not to generate new theoretical categories through inductive thematic analysis, but to use learners’ accounts to contextualize the questionnaire findings from Study 1, examine how learners perceived the extent to which the VR system developed in Study 2 responded to their reported needs, and identify perceived strengths, limitations, and directions for future refinement.

All interview recordings were transcribed verbatim and anonymized using participant codes P1–P10. The 19 interview questions were first mapped onto four predefined analytic modules: prior exposure to CETC in foreign language learning, cultural confidence and foreign-language expression, cross-cultural communication barriers and training needs, and VR learning experience and system refinement needs. These modules provided an initial analytic framework aligned with the research questions and the interview protocol.

Two researchers independently read the transcripts several times and extracted meaning units relevant to the research questions. These meaning units were then organized in an alignment matrix that connected each analytic module with three types of evidence: confirmatory evidence, explanatory evidence, and improvement-oriented evidence. Confirmatory evidence referred to responses that echoed the questionnaire findings, such as learners’ reported lack of confidence in expressing CETC in a foreign language. Explanatory evidence referred to responses that helped explain why specific VR design features were perceived as useful, such as immersive scenarios perceived as helpful for contextualized understanding and object-based interaction perceived as helpful for vocabulary recall. Improvement-oriented evidence referred to comments identifying system limitations or future design needs, such as limited scenario diversity, lack of NPC interaction, insufficient sentence-level scaffolding, or reported motion discomfort.

Disagreements in classification were resolved through discussion until consensus was reached. The final matrix and interpretations were reviewed by the research team to enhance analytic credibility. Because the analysis was descriptive and alignment-oriented rather than designed as quantitative content analysis, statistical inter-coder agreement was not calculated. Representative quotations were selected to illustrate each major finding. The mapping between interview questions and analytic modules is presented in Table S1 of File S2, and the framework-guided qualitative analysis alignment matrix (including confirmatory, explanatory, and improvement-oriented evidence) is provided in Table S2 of File S2. Both are Supplementary Analytical Tables supporting the descriptive qualitative analysis of Study 3.

## 4. Results

### 4.1. Results of Study 1: Learners’ Challenges and Needs

This study examined learners’ challenges in constructing culturally grounded meanings in foreign language use, along with contextual factors related to VR experience and learners’ expectations for VR-supported CETC learning. The results reveal a coherent pattern linking learning challenges, technological readiness, and design-related needs.

#### 4.1.1. Challenges in Constructing Culturally Grounded Meanings

The results identify three interrelated categories of challenges that learners face in constructing culturally grounded meanings in foreign language use: input-related limitations, conceptual-linguistic mapping difficulties, and affective constraints. They were derived through an alignment-based interpretation of the questionnaire content domains and item-level descriptive results, rather than through factor analysis. They summarize learners’ reported difficulties in relation to contextualized cultural input, conceptual-linguistic mapping, and affective readiness for cultural expression.

Table 1 presents the item-level descriptive statistics for all Likert-type items reported in Study 1. All items were scored on a five-point scale ranging from 1 = strongly disagree to 5 = strongly agree. Because all items were completed by the 277 valid participants and shared the same response range, missing values and minimum/maximum values are not separately reported in the table. For each item, the mean, standard deviation, and percentage of participants selecting “agree” or “strongly agree” are reported to provide a transparent description of learners’ perceived challenges, needs, and expectations.

A primary challenge concerns limited access to culturally embedded input in existing foreign language learning materials. As shown in Table 1 and Figure 3, participants generally perceived CETC-related input in existing foreign language learning materials as insufficient (*M* = 3.67, *SD* = 1.06; agree/strongly agree = 61.01%). This perceived lack of cultural input was accompanied by strong learner demand for increased CETC content in foreign language courses (*M* = 4.03, *SD* = 0.91; agree/strongly agree = 77.61%) and interest in CETC-integrated learning (*M* = 4.08, *SD* = 0.90; agree/strongly agree = 78.34%). These findings indicate a mismatch between available instructional resources and learners’ needs for culturally grounded input.

A second challenge lies in the difficulty of transforming cultural understanding into appropriate linguistic expression. Participants strongly endorsed the role of culture in language learning, as indicated by high agreement that foreign language learning involves cultural understanding (*M* = 4.32, *SD* = 0.81; agree/strongly agree = 91.34%) and that CETC knowledge supports comprehension of culturally embedded meanings (*M* = 4.14, *SD* = 0.93; agree/strongly agree = 81.22%). This discrepancy reflects difficulties in mapping culturally grounded meanings onto linguistic forms. At the same time, many participants reported concern about accurately expressing CETC in a foreign language (*M* = 4.01, *SD* = 1.01; agree/strongly agree = 78.34%), suggesting a gap between cultural understanding and foreign-language expression.

#### 4.1.2. Contextual Factors: Learners’ Prior Experience with VR

To contextualize the feasibility of VR-supported learning, participants’ prior experience with VR was examined, including their exposure, usage patterns, and perceptions of its affordances and constraints.

As shown in Figure 4, 45.49% of participants reported prior exposure to VR, while 54.51% had no experience. Among those with experience, engagement levels were generally low: 65.08% reported only brief exposure, and only 7.14% reported frequent use. VR use was predominantly associated with entertainment contexts (67.46%), followed by tourism-related experiences (15.08%), whereas only 14.29% reported using VR for learning purposes. These results indicate limited integration of VR into formal learning contexts.

Among participants with VR learning experience (*N* = 32), both affordances and constraints were reported in the multiple-response survey items. Prevalence percentage is reported below, which reflects the proportion of participants selecting each option and can sum to over 100% for multiple-response questions. As shown in Figure 5A, the most frequently identified advantages were realistic scenarios and strong interactivity (both 71.88%), followed by high interest/engagement (62.5%). Additional perceived benefits included better memory retention (43.75%) and learning efficiency (40.63%). At the same time, several constraints were identified (Figure 5B), including network latency (40.63%), physical discomfort (37.50%), and operational complexity (37.50%). Other concerns included cost (28.13%) and limitations in content realism (25.00%).

Despite limited prior use in educational settings, participants generally reported positive perceptions of VR’s potential for supporting immersive and contextualized learning (see Figure 6). These perceptions provide a contextual basis for considering VR as a potential medium for addressing the challenges identified above.

#### 4.1.3. Learner Expectations for VR-Supported CETC Learning

Learners’ expectations for a CETC-integrated VR system further reflect their perceived needs in addressing the challenges identified in Section 4.1.1.

Participants reported strong expectations for features that support culturally grounded meaning construction. A large majority (*M* = 4.29, *SD* = 0.75; agree/strongly agree = 89.17%) expected the system to provide immersive cultural scenarios, indicating a need for context-rich input environments. Additionally, 91.70% (*M* = 4.32, *SD* = 0.73) emphasized the importance of speech recognition for supporting language production, suggesting demand for tools that facilitate the mapping of meaning to linguistic expression.

Learners also highlighted the importance of personalization, with 91.33% (*M* = 4.40, *SD* = 0.73) expecting adaptive difficulty and 93.50% (*M* = 4.39, *SD* = 0.69) requesting personalized feedback. These expectations point to the need for scaffolding mechanisms that accommodate varying levels of linguistic and cultural competence.

Participants demonstrated strong willingness to adopt CETC-integrated VR-supported learning systems (Figure 7), with 80.87% (*M* = 4.08, *SD* = 0.81) indicating willingness for sustained use and 83.39% (*M* = 4.14, *SD* = 0.82) expressing readiness to participate if such courses were offered.

In terms of expected outcomes, 80.51% (*M* = 4.10, *SD* = 0.85) anticipated improved confidence in cross-cultural communication, and 87.37% (*M* = 4.24, *SD* = 0.75) expected enhancement in intercultural communicative competence. Additionally, a large proportion reported willingness to express (*M* = 4.19, *SD* = 0.82; agree/strongly agree = 83.75%) and disseminate (*M* = 4.31, *SD* = 0.76; agree/strongly agree = 89.90%) CETC through foreign language use.

Participants emphasized the importance of opportunities for culturally grounded communication. Specifically, 84.47% (*M* = 4.18, *SD* = 0.79) highlighted the need for increased opportunities to practice expressing CETC in a foreign language. These expectations indicate a demand for learning environments that support both the development of cultural conceptual knowledge and its application in communicative contexts.

### 4.2. Results of Study 2: Design of the VR-Based Learning System

#### 4.2.1. Translation of Theoretical Principles into Design Features

The results of Study 2 demonstrate how abstract theoretical principles were systematically translated into concrete design features of the VR-based language learning system, thereby addressing RQ2.

From the perspective of cultural linguistics, the principle of language–culture symbiosis was operationalized through the embedding of linguistic elements within culturally meaningful contexts. Specifically, CETC-related vocabulary and expressions (e.g., “red envelope”, “zongzi”, “tuanyuan”) were not presented in isolation but were integrated into interactive cultural scenarios such as festival participation and daily practices (Figure 8). This design is intended to enable learners to learn linguistic forms alongside their underlying cultural conceptualizations, rather than as decontextualized symbols.

The principle of cultural conceptual alignment was reflected in the selection and organization of content. The system incorporated representative CETC domains, including major traditional festivals and symbolic cultural practices, ensuring that learning materials authentically reflect core cultural values such as collectivism and harmony. This content design directly responds to the need for culturally grounded meaning construction identified in Study 1, and its on-site implementation is shown in Figure 9.

From the perspective of multimedia affective–cognitive theory, the modality principle was realized through the integration of multimodal input channels, including visual (immersive VR scenes), auditory (native-speaker pronunciation), and textual (bilingual annotations) information. This multimodal configuration was designed to optimize cognitive processing and reduce extraneous cognitive load.

In addition, the emotional design principle was operationalized through the creation of affectively engaging scenarios (e.g., festive celebrations and ritual activities), while the interaction principle was implemented via task-based and embodied interactions such as object manipulation and scenario-based communication. Together, these features support both cognitive integration and affective engagement in the learning process.

#### 4.2.2. Design Responses to Identified Learning Challenges

Building on the findings of Study 1, the system design addressed three major categories of learner-reported challenges: insufficient contextualized CETC input, difficulties in mapping cultural meanings onto linguistic forms, and affective barriers. These design responses should be understood as prototype-level solutions rather than as evidence that these challenges were empirically resolved.

First, to respond to the lack of contextualized CETC input, the prototype introduced immersive festival scenes and culturally meaningful objects, allowing learners to encounter language within visual and cultural contexts. Second, to address meaning-mapping difficulties, the system incorporated object-based vocabulary learning and structured dialogue practice, helping learners connect target-language expressions with cultural objects, practices, and meanings. Third, to respond to affective barriers such as nervousness and low communicative confidence, the system provided scaffolded prompts, repeatable practice, auditory replay, and formative feedback, with the aim of creating a relatively low-risk environment for language practice.

In this way, Study 2 translated the needs-analysis findings into prototype-level system features. The effectiveness of these features in improving language production, reducing anxiety, or enhancing intercultural communicative competence requires further empirical testing.

#### 4.2.3. Core System Features Supporting Meaning Construction

The system’s support for culturally grounded meaning construction is operationalized through three core functional modules, each of which integrates a specific learning problem, design function, and theoretical foundation.

The Cultural Content Module is designed to address the problem of fragmented and decontextualized cultural knowledge in traditional language learning. It provides structured CETC content centered on key cultural domains, including major festivals and symbolic practices. Interactive elements enable learners to explore cultural meanings embedded in these contexts. Grounded in cultural linguistics, this module is intended to support the construction of culturally situated semantic representations.

The Language Skill Module is designed to respond to the challenge of disconnect between linguistic forms and meaningful use. It integrates listening, reading, and speaking skills within CETC contexts through multimodal input and output mechanisms, including pronunciation playback, bilingual support, and speech recording with feedback. Based on the modality principle and cognitive load theory, this module is intended to facilitate the integration of linguistic processing and meaning construction.

The Situational Immersion Module is designed to address the lack of experiential learning opportunities in conventional instruction. It creates immersive VR environments with real-time interaction and scenario-based tasks, allowing learners to participate in culturally situated activities. Drawing on principles of embodied interaction and experiential learning, this module is intended to enhance contextualized understanding and support deeper cognitive–affective engagement.

These three modules work together within each scenario. For example, in a Spring Festival scene, the Cultural Content Module provides culturally meaningful objects and practices, the Language Skill Module supports vocabulary, pronunciation, and dialogue practice, and the Situational Immersion Module embeds these learning activities within an interactive exchange with a virtual foreign peer. In this way, cultural understanding, language use, and embodied interaction are integrated within a single learning sequence.

#### 4.2.4. Technical and Interaction Design Outcomes

The technical implementation of the system reflects a series of design decisions aimed at enhancing usability, accessibility, and interaction quality. The system was developed using Unity 3D and deployed on the Meta Quest 3 platform, which supports standalone operation without external hardware. This hardware selection directly responds to usability concerns identified in Study 1, particularly the need to reduce technical complexity and increase accessibility. In addition, several design strategies were implemented to mitigate motion sickness and physical discomfort, including stable frame rates (approximately 60 fps), smooth navigation transitions, and reduced abrupt visual shifts. These optimizations are intended to contribute to a more comfortable and sustainable immersive learning experience. Finally, the system incorporates user-centered interaction features, such as intuitive object selection, responsive feedback, and adjustable interface elements (e.g., text size and interaction timing). These design outcomes are collectively intended to enhance the usability and practicality of the VR-based learning system.

### 4.3. Results of Study 3: Learners’ Perceptions and Experiences of VR-Supported Meaning Construction

#### 4.3.1. Baseline Learning Experiences and Limitations in Cultural Integration

Participants reported that CETC was present in their foreign language learning experiences through multiple channels, with textbooks and classroom instruction being the most common sources. However, such cultural content was typically presented in a fragmented and unsystematic manner, often limited to specific themes such as traditional festivals or exam-related materials. Classroom integration was generally supplementary, relying on brief teacher explanations, isolated examples, or short video clips. For example, P1 noted that “the textbook did not really include intangible cultural heritage, and in class we only watched some English expressions about Spring Festival or Lantern Festival customs”. Similarly, P4 stated that cultural content usually appeared “in reading passages, exam materials, or short videos, rather than as a complete learning context”. These comments suggest that CETC-related content was present in learners’ prior experience, but often in a fragmented and supplementary form.

Despite this limitation, participants generally perceived CETC as a motivational resource. They reported that familiar cultural content made foreign language learning more meaningful and closer to life. As P4 explained, “Learning English through content I am interested in makes English feel closer to life and motivates me to continue learning”. P3 similarly expressed the view that integrating Chinese traditional culture into English learning might help learners build cultural confidence. These responses support the findings of Study 1 by showing that learners valued CETC integration, while also highlighting the need for more systematic and context-rich cultural representation.

#### 4.3.2. Learners’ Challenges in Culturally Grounded Language Use

Participants consistently described a gap between cultural knowledge and foreign-language expression. Many learners expressed confidence in Chinese culture itself but lacked confidence in expressing culturally specific meanings accurately in a foreign language. P1 stated, “I know a lot about Chinese traditional culture, but I usually cannot express it in a foreign language”. P5 similarly noted, “I am very confident in the culture, but I become nervous because my vocabulary is not enough”. P7 described this experience as a sense of “not being able to express exactly what I want to say”, explaining that the words available to them often failed to convey the intended cultural meaning.

The main barriers included insufficient vocabulary, unfamiliar cultural terminology, limited sentence patterns, weak oral fluency, and concerns about inaccurate expression. For example, P10 recalled difficulty introducing a specific cultural location in Suzhou because they “could not express the place accurately in English” and had to rely on general descriptions. These comments indicate that learners’ difficulties were not only linguistic but also conceptual and expressive. They often knew the cultural meaning in Chinese but struggled to recontextualize it in a foreign language.

Affective factors also shaped learners’ willingness to communicate. Several participants reported nervousness in public or unfamiliar communicative situations, especially when they were concerned about making mistakes or misrepresenting cultural meanings. These findings echo the questionnaire results from Study 1 and further clarify that the key challenge lies in coordinating cultural knowledge, linguistic resources, and communicative confidence.

#### 4.3.3. Cross-Cultural Communication Needs and Training Expectations

Participants also reported difficulties in cross-cultural communication more broadly. Common barriers included fast speech, unfamiliar accents, limited vocabulary, delayed response, lack of topics, and cultural cognitive differences. P2 explained, “First, I may not understand what the other person says; even if I understand it, I may not have a good sentence pattern to respond”. P1 similarly evaluated their “cross-cultural expression ability” as weak while describing their understanding ability as relatively better.

When asked about preferred training methods, participants expressed strong interest in role play, simulated dialogue, and scenario-based practice. P3 stated that “scenario-based dialogue would be the most effective”, while P7 emphasized that role play and interactive scenarios could create a stronger sense of participation. These responses indicate that learners expected more than cultural knowledge input; they wanted opportunities to practice culturally situated communication in realistic contexts.

Participants’ expectations also pointed to future system development needs. Several participants suggested adding daily-life communication, table manners, festival interaction, academic exchange, NPC dialogue, and even multiplayer interaction. P6 suggested that the system “could be like artificial intelligence and have simple conversations with me; if I make mistakes, it could correct me”. P10 similarly suggested that “daily conversations, such as asking for directions or talking with friends”, would be especially useful. These findings provide improvement-oriented evidence for refining the system from object-based learning toward more dialogic and socially interactive communication.

#### 4.3.4. Learners’ Experiences of VR-Supported Meaning Construction

Participants generally evaluated the VR system positively and described it as novel, immersive, and engaging. P1 commented that the system was “very realistic”, noting that the clouds and fireworks in the Spring Festival scene were clear and vivid. P10 described the experience as “very immersive and realistic”, while P8 stated that VR allowed them to “learn in a scene” and encounter things that were difficult to learn from textbooks alone.

The interviews also explained why VR was perceived as useful for culturally grounded meaning construction. Participants repeatedly mentioned that VR connected words with objects, scenes, and cultural practices. P4 stated that VR “turns the words into concrete objects, and that makes them more memorable”. P8 similarly explained that when a word appeared together with an image, learning became “less rigid” than textbook-based instruction. These responses suggest that learners perceived object-based and scene-based interaction as helpful for aligning linguistic forms with cultural meanings.

In addition, learners perceived VR as providing a more active and low-risk learning environment. P9 noted that the system “gives the initiative to the learner; you can choose what you want to learn and listen to”. P7 stated that learning through VR was “more interesting” than ordinary classroom learning and that they were more willing to learn in this way. Several participants also expected that long-term VR use might help reduce nervousness in real communication by providing simulated practice in culturally meaningful environments.

#### 4.3.5. Overall Evaluation and Design Expectations

Participants identified several strengths of the current system, including immersion, visual realism, dynamic scene elements, object interaction, multilingual support, and ease of operation after initial familiarization. Specific features such as fireworks, flowing water, falling leaves, teleportation, and clickable vocabulary labels were frequently mentioned as memorable and engaging.

At the same time, participants also identified limitations and areas for improvement. The most common suggestion was to add more NPCs, and more dialogue-based interaction. P1 commented that “there were only stalls but no people, which felt a little strange”. P2 stated that the system should add more “NPCs for communication”. Other suggestions included adding sentence-level scaffolding, phrase decomposition, phonetic support, more cultural background explanations, richer scenes, larger spaces, smoother operation, and more precise object clicking.

A small number of participants also mentioned physical or usability concerns. P2 reported that the headset felt heavy, while P9 experienced slight dizziness, and P10 reported dizziness and nausea that led to ending the experience early. These comments indicate that although most interviewed participants reported no discomfort, physical comfort and usability remain important considerations for future system refinement.

Overall, the interview findings indicate that learners perceived VR not merely as a technological tool, but as a potentially integrative learning environment that could connect cultural context, linguistic expression, and emotional engagement. At the same time, learners’ feedback suggests that future versions should add richer social interaction, sentence-level language support, and more adaptive communicative scaffolding.

To clarify the alignment across the three studies, Table 2 summarizes how the major challenges and needs identified in Study 1 informed the prototype design features in Study 2 and how these features were perceived by learners in Study 3. Table S3 in File S2 summarizes the core thematic findings of Study 3 with representative participant quotations.

## 5. Discussion

This study provides converging evidence that integrating CETC into foreign language learning requires not only enriched cultural input but also the alignment of cognitive, linguistic, and affective processes. Study 1 revealed a systematic mismatch between learners’ strong demand for CETC-related expression and the limited, fragmented cultural input in existing curricula. It further identified three interrelated challenges: insufficient contextualized input, difficulties in mapping cultural meanings to linguistic forms, and affective barriers such as anxiety and low communicative confidence. Study 2 addressed these challenges by developing a VR-based learning system grounded in cultural linguistics and cognitive–affective principles, embedding CETC into immersive, interactive contexts. Study 3 provided qualitative evidence that learners perceived the system as potentially supportive of contextualized understanding, aligned language expression, and lowered communication anxiety. These findings suggest that culturally grounded meaning construction is not a purely linguistic process but a cognitive–affective–contextual process requiring the alignment of cultural conceptual knowledge, linguistic resources, and affective readiness. VR, in this context, functions as a powerful medium for facilitating such alignment by integrating immersive input, situated interaction, and emotional support.

### 5.1. Discussion of Study 1: Learners’ Challenges and Needs

The findings of Study 1 reveal that learners’ difficulties in constructing culturally grounded meanings are not limited to linguistic proficiency, but rather emerge from the interaction between insufficient cultural input, challenges in conceptual–linguistic mapping, and affective constraints (Z. Liu, 2022; Mardones et al., 2024). First, the reported lack of CETC-related content in existing instructional materials (61.01%) confirms a persistent imbalance in foreign language education, where cultural learning remains peripheral to linguistic skill development. This aligns with previous research indicating that language instruction often prioritizes functional communication while neglecting culturally embedded meaning systems (Bailey et al., 2025; Harman & Jin, 2022; Hossain, 2024; S.-H. Wang et al., 2021). From the perspective of cultural linguistics, meaning construction depends on learners’ access to culturally grounded conceptual schemas, which are typically underrepresented in decontextualized learning materials (Bian, 2021; Mardones et al., 2024; Shi & Ma, 2025). Therefore, the observed mismatch between high learner demand (over 77%) and limited instructional provision suggests that current pedagogical approaches fail to support the development of culturally situated meaning-making processes (Bian, 2021; Ntumi et al., 2026; S.-H. Wang et al., 2021).

In addition to input limitations, the findings highlight a critical but underexplored challenge: the difficulty of mapping cultural conceptualizations onto linguistic forms. Although learners demonstrated strong awareness of the role of culture in language learning (over 90%), a substantial proportion (78.34%) reported difficulty in expressing CETC in a foreign language. This discrepancy reflects a gap between conceptual knowledge and linguistic realization, which cannot be fully explained by vocabulary deficits alone (Mondal, 2024). Instead, it points to the absence of structured opportunities for integrating cultural meaning with language production (Cong-Lem, 2025; Hossain, 2024; Xu et al., 2025). This finding extends prior research on ICC, which has emphasized knowledge and attitudes, by underscoring the importance of proceduralizing cultural knowledge into communicative practice (Zhou et al., 2024). From a cognitive perspective, this process may impose high intrinsic and germane cognitive load, particularly when learners must simultaneously manage cultural interpretation and linguistic encoding. The lack of scaffolding mechanisms in traditional language learning environments may further exacerbate this difficulty.

Importantly, Study 1 also demonstrates that the challenges in culturally grounded meaning construction are deeply intertwined with affective factors, particularly learners’ confidence in cross-cultural expression. The high level of concern regarding CETC expression (78.34%) suggests that learners’ communicative willingness is constrained not only by competence but also by perceived risk and anxiety. This finding resonates with the affective filter hypothesis (Krashen, 1982) and recent research on language anxiety in intercultural contexts (Lu, 2025; Z. Qu & Chen, 2026), which emphasize that emotional factors can inhibit the activation of existing knowledge. In this regard, the generally positive perceptions of VR, despite limited prior exposure, are particularly noteworthy. Learners’ expectations for immersive scenarios (89.17%), speech-based interaction (91.70%), and personalized feedback (93.50%) indicate a demand for learning environments that can simultaneously provide contextualized input, scaffolded output, and affective support. Taken together, these findings not only clarify the multidimensional nature of learners’ challenges (RQ1), but also provide a theoretically grounded rationale for the design of VR-supported learning environments in Study 2, particularly in terms of reducing cognitive load, supporting meaning–form mapping, and lowering affective barriers.

### 5.2. Discussion of Study 2: Design of the VR-Based Learning System

Rather than treating VR as a purely technological enhancement, this study conceptualizes system development as a process of translating cognitive, linguistic, and affective principles into pedagogically meaningful design features. This perspective aligns with previous research on immersive technologies, which emphasizes that VR environments simultaneously engage users’ cognitive processing, emotional responses, and behavioral interactions, thereby shaping learning outcomes in a more integrated manner (Raman et al., 2025; Rudling et al., 2026; Ruscica et al., 2025). By foregrounding these interrelated dimensions, the present study moves beyond technology-driven approaches and highlights the importance of designing learning environments that support coordinated cognitive and affective engagement. In particular, the integration of cultural linguistics into system design represents a critical advancement over previous VR-based language learning systems, such as Mondly, Language Lab, and ImmerseMe, which have predominantly focused on situational immersion without explicitly modeling the role of cultural conceptual systems (Dhieb, 2024; Hajizadeh et al., 2024). By embedding CETC-related linguistic forms within culturally meaningful scenarios, the system operationalizes the principle of language–culture symbiosis, enabling learners to construct meaning through interaction with culturally grounded contexts rather than through decontextualized input. In addition, this design also extends beyond system-level innovation to address a broader pedagogical challenge identified in prior research, namely, the persistent disconnection between language instruction and learners’ lived cultural environments (Shi & Ma, 2025). Existing studies on place-based and culturally responsive education have consistently shown that insufficient integration of local cultural knowledge, coupled with limited teacher capacity and resource constraints, leads to reduced learner engagement and weakened cultural identity formation (Petri et al., 2025; Shi & Ma, 2025). Hence, the VR system developed in the present study can be understood not merely as a technological tool, but as a mechanism for reconfiguring how cultural knowledge is integrated into language learning, shifting it from a curriculum-dependent practice to an experience-driven, system-embedded process.

Furthermore, the design of interactive tasks and multimodal input mechanisms offers a targeted response to the meaning–form mapping challenge identified in Study 1. While previous research on intercultural communicative competence has emphasized the acquisition of cultural knowledge, relatively little attention has been paid to how such knowledge is transformed into communicative practice (Z. Qu & Chen, 2026; Zhou et al., 2024). The present system is designed to address this gap by integrating role-based interaction, object manipulation, and scenario-driven communication tasks, which are collectively intended to facilitate the alignment between cultural conceptualizations and linguistic expression. According to Cognitive Load Theory and the Cognitive Theory of Multimedia Learning, immersive VR environments present a complex cognitive context in which learners must allocate limited processing resources across multiple sensory channels and interactive demands (De Witte et al., 2024, 2025; C. Song et al., 2023). While multimodal input (e.g., visual, auditory, and textual information) has the potential to support meaning construction, prior research has also shown that such environments may increase cognitive load due to heightened attentional demands and potential redundancy across information sources (Bali et al., 2026; Rudling et al., 2026). Against this backdrop, the present system does not assume that multimodality or immersion inherently reduces cognitive load; rather, it adopts a design approach aimed at managing and structuring learners’ cognitive processing within immersive environments. Specifically, the integration of role-based interaction, object manipulation, and scenario-driven tasks is designed to guide attention toward task-relevant information, while minimizing unnecessary processing demands associated with unstructured exploration or redundant input. In this sense, structured interaction is intended to function as a form of cognitive scaffolding, intended to help learners coordinate cultural conceptualizations with linguistic expression without overwhelming their processing capacity. This perspective moves beyond the assumption that immersion alone is sufficient for learning, emphasizing instead that the effectiveness of VR-based language learning depends on how cognitive load is regulated through principled instructional design rather than merely reduced.

In addition to cognitive and linguistic considerations, the system design explicitly addresses learners’ affective barriers, thereby reinforcing the role of emotional factors in culturally grounded communication. The incorporation of adaptive scaffolding, dual-mode interaction (guided vs. immersive), and low-risk virtual environments reflects an intentional effort to reduce anxiety and enhance communicative confidence, consistent with the affective filter hypothesis and recent research on emotion in language learning (Ding & Yusof, 2025; X. Wang & Zhou, 2025; H. Yan & Singh, 2026). Notably, the inclusion of user-centered technical optimizations, such as reduced latency, improved usability, and mitigation of motion sickness, further supports sustained engagement, aligning with a growing body of research that emphasizes the importance of usability and physiological comfort in immersive learning environments, yet still often lacks systematic integration into pedagogically driven VR design (Geris et al., 2024; Z. Zhang et al., 2025). Taken together, these findings suggest that the effectiveness of VR-based language learning does not derive from immersion per se, but from the alignment between theoretical principles, learner needs, and system affordances. This design-oriented contribution not only provides a concrete response to RQ2, but also offers a replicable framework for developing culturally grounded VR learning environments, thereby bridging the gap between abstract theory and practical implementation in educational technology research.

### 5.3. Discussion of Study 3: Learners’ Perceptions and Mechanisms of VR-Supported Meaning Construction

The findings of Study 3 provide qualitative evidence for the learning challenges identified in Study 1, while offering further insight into how these challenges are experienced and negotiated by learners in prior learning experiences and simulated VR-supported contexts. Although the interview participants were not necessarily drawn from the same sample as Study 1, similar challenges were articulated, suggesting that these issues may be relevant across different learner groups. Consistent with previous research, participants described how cultural content was often experienced as fragmented and decontextualized, which they perceived as constraining their ability to construct coherent cultural meanings during communication (Shi & Ma, 2025; Zhou et al., 2024). Rather than merely identifying these challenges, learners elaborated on the process through which they struggled to translate cultural understanding into linguistic expression, reflecting a persistent meaning-construction gap. This gap, coupled with linguistic limitations and affective constraints, was experienced as difficulty in coordinating multiple demands in real-time communication (Hoemann et al., 2025; Yin, 2026). Crucially, Study 3 captures how these challenges are subjectively experienced by learners and further shows that learners perceived the VR environment as supporting their sense-making processes when engaging with cultural content. This shift from theoretical design rationale to learner-perceived functionality provides an important layer of learner-perspective evidence, suggesting that VR-based systems should be examined not only in terms of design features, but also in terms of how these features are interpreted and utilized by learners in practice (Hickethier et al., 2025; Z. X. Zhang, 2026).

More specifically, the findings illustrate that learners perceived the VR system as supporting contextual understanding, expressive alignment, and emotional engagement. First, immersive environments were perceived as providing context-rich and multimodal cultural input, helping learners move beyond abstract representations toward more situated understanding. This is consistent with theoretical claims from situated learning and multimedia learning, but also suggests that such affordances are meaningful only when learners perceive them as relevant and usable within specific tasks (Makransky & Petersen, 2021; Scherr et al., 2025; Skulmowski et al., 2016). Second, VR was perceived as potentially facilitating language practice in context, particularly through scenario-based interaction and task-oriented engagement. From the learners’ perspective, these features were perceived as helping reduce the cognitive distance between conceptual understanding and language production, thereby offering a possible way to address the meaning–form mapping challenge identified in Study 1. This finding extends prior research on intercultural communicative competence by highlighting that the transformation of cultural knowledge into communicative practice requires not only knowledge exposure but also structured opportunities for contextualized use (Wibowo et al., 2024; Zhou et al., 2024). Third, the affective dimension emerged as a critical enabling condition. Participants described the VR environment as a relatively low-risk space that might help reduce perceived anxiety and increase willingness to communicate. Unlike the design assumptions outlined in Study 2, Study 3 suggests that such affective affordances are not merely embedded in the system, but are actively perceived and experienced by learners as facilitating engagement. This highlights the central role of emotional safety in sustaining engagement.

At the same time, the interview findings also reveal important boundaries of the current system. The most frequently mentioned improvement was the need for richer social interaction, including NPCs, dialogue-based tasks, and sentence-level scaffolding. Some participants also suggested expanding the range of daily-life and festival-based communication scenarios, while a few reported usability or physical-comfort issues such as headset weight or dizziness. These comments indicate that culturally grounded meaning construction in VR requires not only immersive cultural representation, but also sustained communicative interaction and adaptive linguistic support.

Taken together, the findings of Study 3 indicate that learners do not perceive VR as merely a technological apparatus, but rather as an integrative learning environment that mediates the interaction between input, meaning construction, and affective engagement. This perception is of particular significance, as it signifies a shift from technology-centred to learner-centred understandings of immersive learning. While Study 2 conceptualised VR as a system that embeds cognitive, linguistic, and affective principles into design, Study 3 suggests that the perceived usefulness of such integration depends on whether learners experience these elements as coherent and supportive of their learning processes. Furthermore, the results of the study suggest that the potential educational value of VR does not lie in immersion per se, but in its perceived capacity to help bridge persistent gaps in language learning. These gaps primarily refer to the disconnection between cultural knowledge and expression, and the tension between communicative demands and affective constraints. Importantly, these findings reframe such gaps not merely as static learning difficulties, but as dynamically experienced tensions that may be addressed through appropriately designed learning environments. By foregrounding learners’ perceptions and experiences, this study contributes to a more nuanced understanding of how immersive technologies may function as meaning-making environments, thereby extending existing research on VR-assisted language learning beyond questions of effectiveness toward questions of mechanism and learner experience.

### 5.4. Limitations

Several limitations should be acknowledged. First, the evidentiary scope of this study is limited. The study provides needs-analysis data, a theory-informed VR system design, and exploratory learner-perception evidence based on a short VR experience, but it does not directly test learning effectiveness. Specifically, it did not include pre–post measures, objective language-production data, retention or transfer tasks, assessment of intercultural communicative competence, or a comparison/control condition. Therefore, the findings should be interpreted as evidence of learner needs, design feasibility, and learner-perceived usefulness rather than as evidence of learning gains. Future research should employ controlled, longitudinal, or classroom-based intervention designs to examine whether CETC-integrated VR learning environments lead to measurable improvements in language production, culturally grounded meaning construction, retention, transfer, and intercultural communicative competence. Second, the questionnaire used in Study 1 was designed as a needs-analysis instrument rather than as a standardized psychometric scale. Although it was informed by relevant literature, reviewed for content relevance, and pilot-tested before formal administration, its Likert-type items were intended to capture heterogeneous learner needs, perceptions, and expectations rather than validated latent constructs. Future studies could develop and validate dedicated instruments for assessing culturally grounded meaning construction in foreign language learning. Third, Study 3 was exploratory and formative in nature. Participants experienced the VR system for only a short period, and the formal qualitative analysis was based on interviews with 10 participants. The findings may therefore reflect immediate impressions and possible novelty effects associated with short-term VR use. In addition, the qualitative analysis was framework-guided and descriptive rather than designed to generate a new theory. Future research should include larger and more diverse interview samples, longer exposure periods, repeated learning sessions, and delayed follow-up assessments. Fourth, the generalizability of the findings is limited. The participants were university students in the Chinese higher education context, and the system content focused mainly on selected CETC scenarios, especially traditional festivals and representative cultural symbols. Learners from different educational levels, language backgrounds, cultural contexts, or levels of prior VR experience may respond differently. Finally, the current VR system should be understood as a prototype rather than a fully developed instructional platform. Its existing functions mainly support immersive scene exploration, pre-scene reading, object-based vocabulary learning, structured dialogue practice, auditory input, oral imitation, and formative AI-based scoring. The AI-based oral-scoring function should also be interpreted only as formative practice feedback. Because the prototype relied on a third-party proprietary speech assessment service, and the scoring procedure was not validated against teacher ratings, standardized language tests, or expert-assessed language performance, the oral scores should not be treated as objective evidence of language-learning outcomes. In addition, the current prototype provided predefined learner-selected difficulty modes rather than automatic performance-based adaptivity; it did not automatically adjust task difficulty, learning content, or feedback based on learner performance. The system does not yet provide fully open-ended communication, advanced natural language interaction, or comprehensive adaptive feedback. Future development should further enhance the system’s interactional depth, linguistic scaffolding, usability, physical comfort, scoring validation, automatic adaptivity, privacy safeguards, and pedagogical integration before its effectiveness is tested in formal instructional contexts.

## 6. Conclusions

Through a multi-phase design-based research approach, this study identified learners’ challenges in expressing CETC in foreign languages, developed a theoretically grounded VR-assisted learning system, and explored learners’ perceptions and experiences of engaging with the system. The findings suggest that learners’ difficulties are not solely linguistic in nature, but emerge from the interaction between insufficient contextualized cultural input, challenges in aligning cultural meanings with linguistic forms, and affective constraints such as anxiety and low communicative confidence. In response, the VR-based learning system was designed to integrate culturally situated scenarios, multimodal input, embodied interaction, and adaptive scaffolding mechanisms to support meaning construction within immersive environments. Our results suggest that the potential educational value of VR may not derive from immersion alone, but from its potential capacity to mediate the relationship between cultural conceptual knowledge, language expression, and affective engagement. Learners perceived the VR environment as potentially facilitating contextualized understanding, expressive alignment, and low-risk communication practice. These findings contribute to existing research on intercultural communicative competence and VR-assisted language learning by foregrounding culturally grounded meaning construction as a dynamic cognitive-affective-contextual process. Overall, this study provides preliminary evidence of feasibility, learner-perceived usefulness, and design value, and offers a theoretically informed and practice-oriented framework for integrating local cultural resources into immersive language learning environments. Future studies are needed to examine the learning effectiveness of such environments through objective outcome measures, comparison conditions, and longer-term intervention designs.

## Figures and Tables

**Figure 1:**
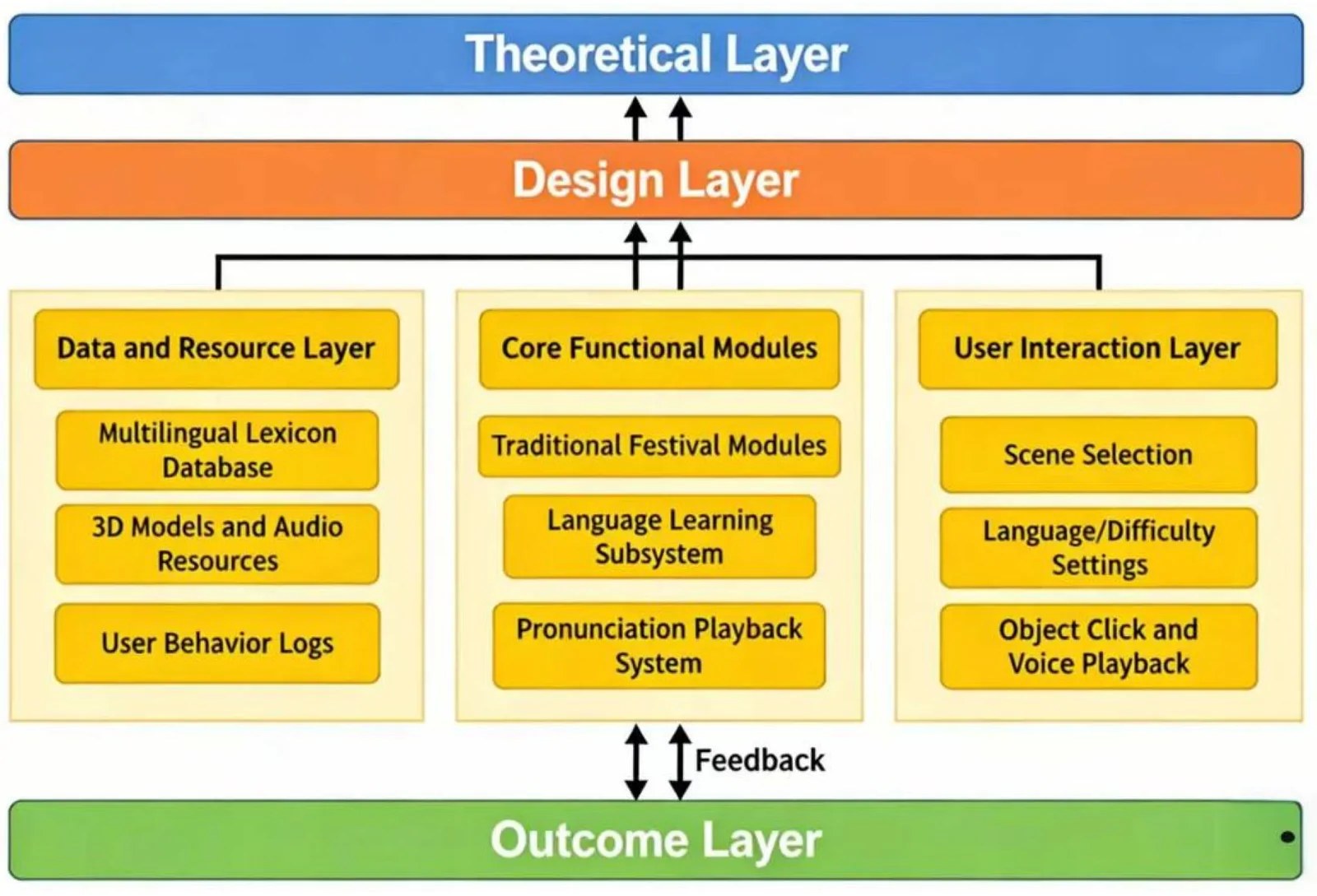
Three-Tier Conceptual Framework of the CETC-Integrated VR Foreign Language Learning System.

**Figure 2:**
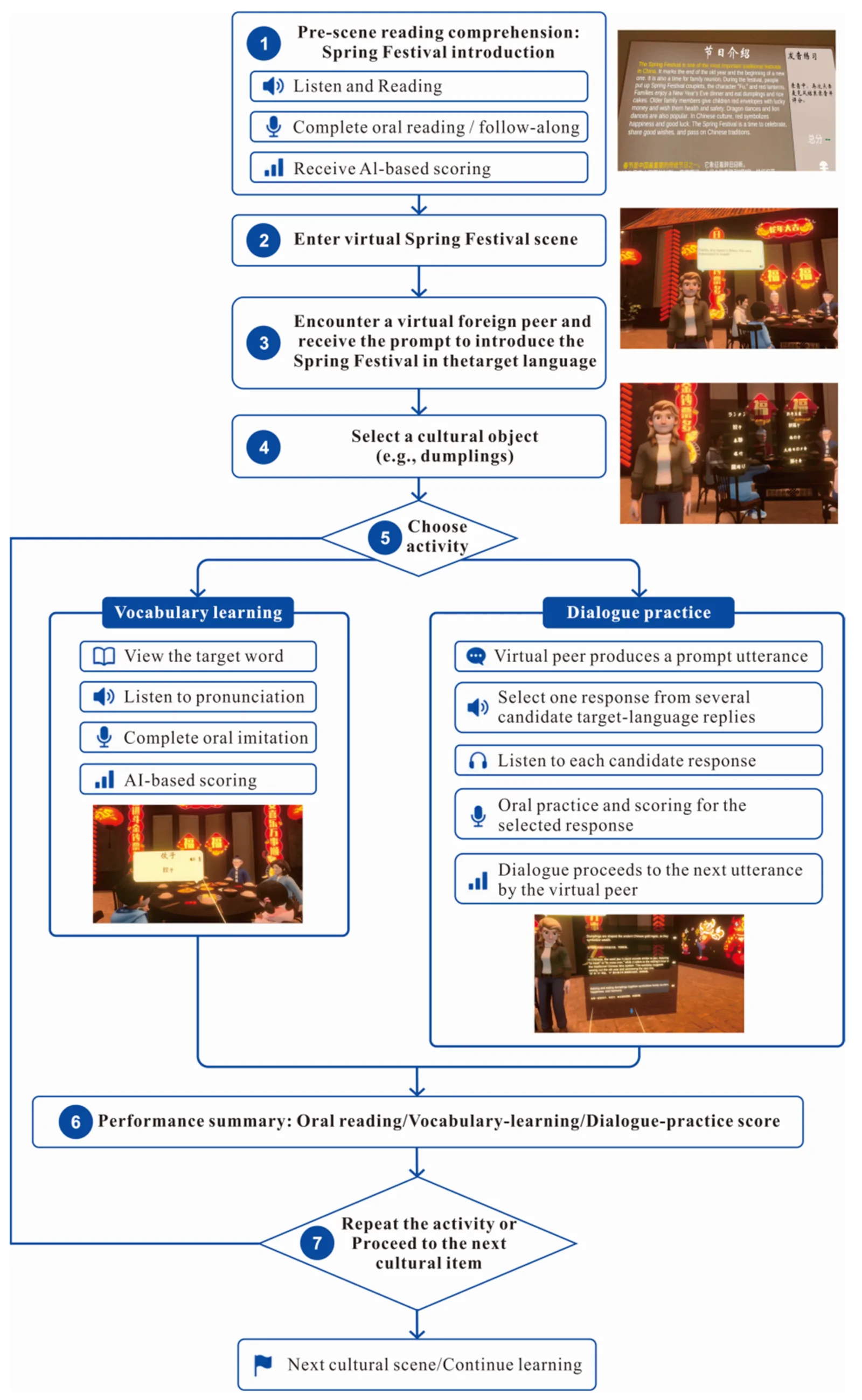
Example Interaction Flow in the Spring Festival Scenario.

**Figure 3:**
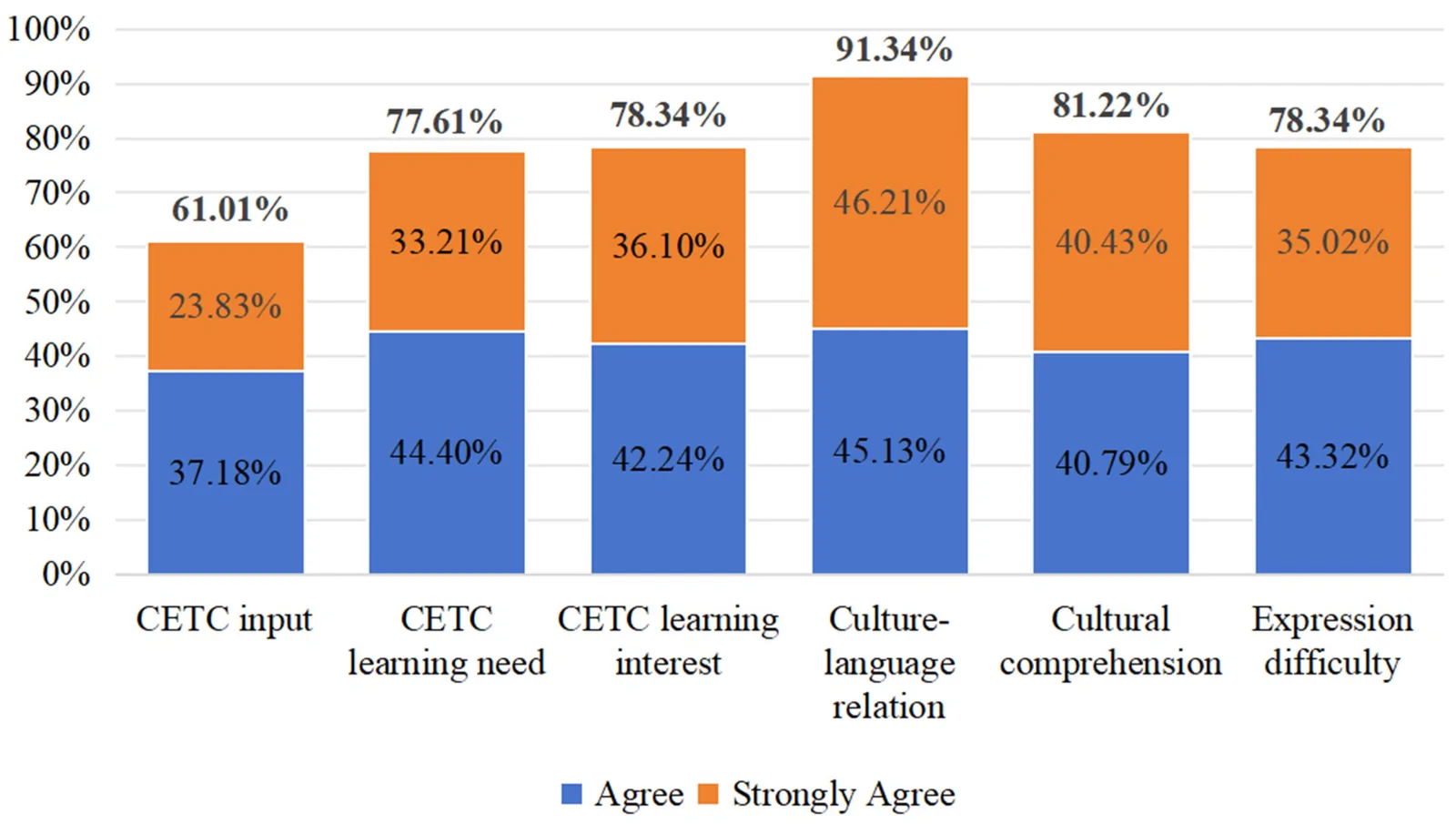
Students’ Perception and Demand for CETC Content.

**Figure 4:**
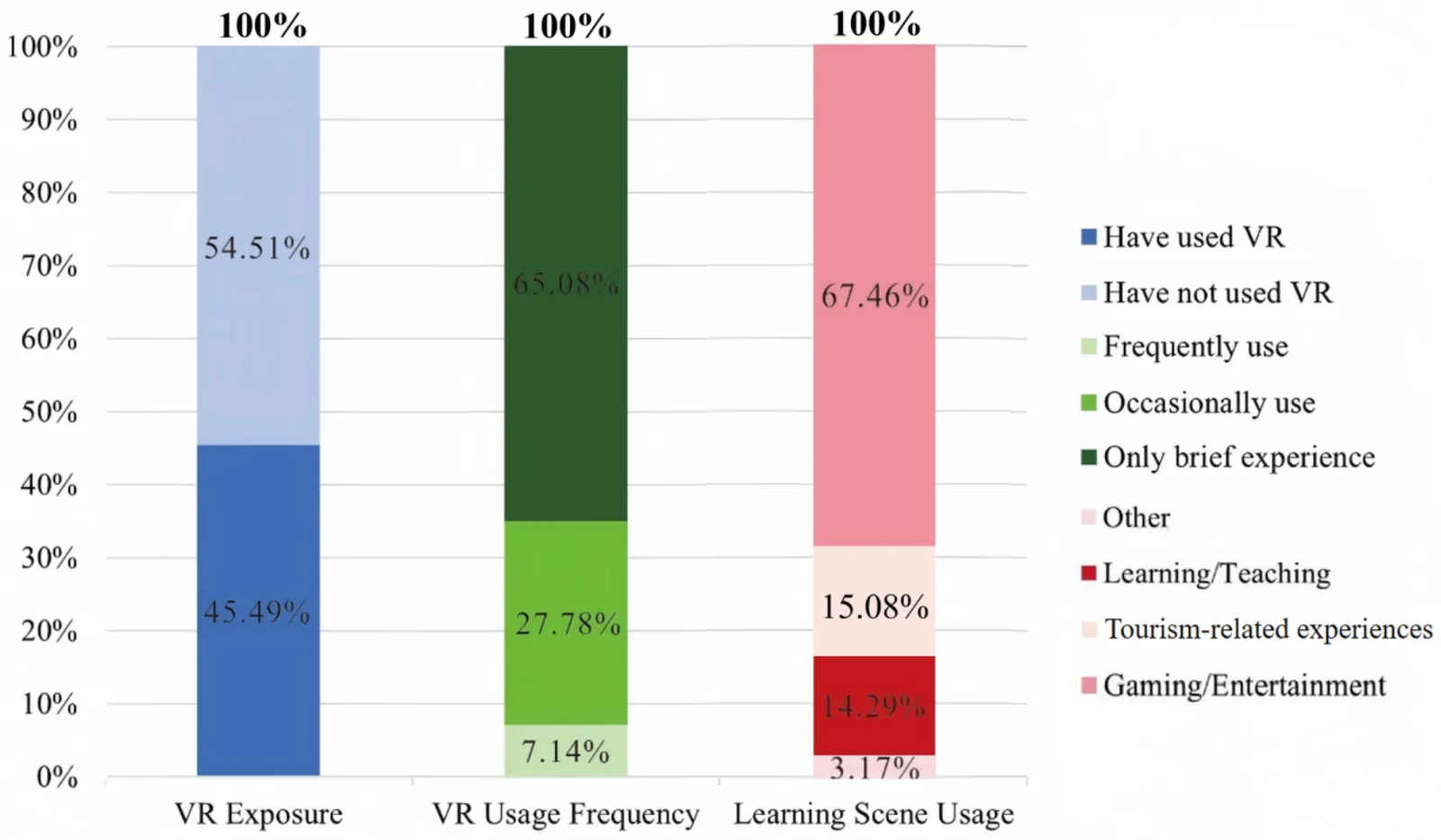
VR Technology Usage and Learning Scene Penetration. VR Technology Usage and Learning Scene Penetration. The percentages in the first column were calculated based on the total sample (*N* = 277). The percentages in the second and third columns were calculated among participants who had previous VR experience (*N* = 126).

**Figure 5:**
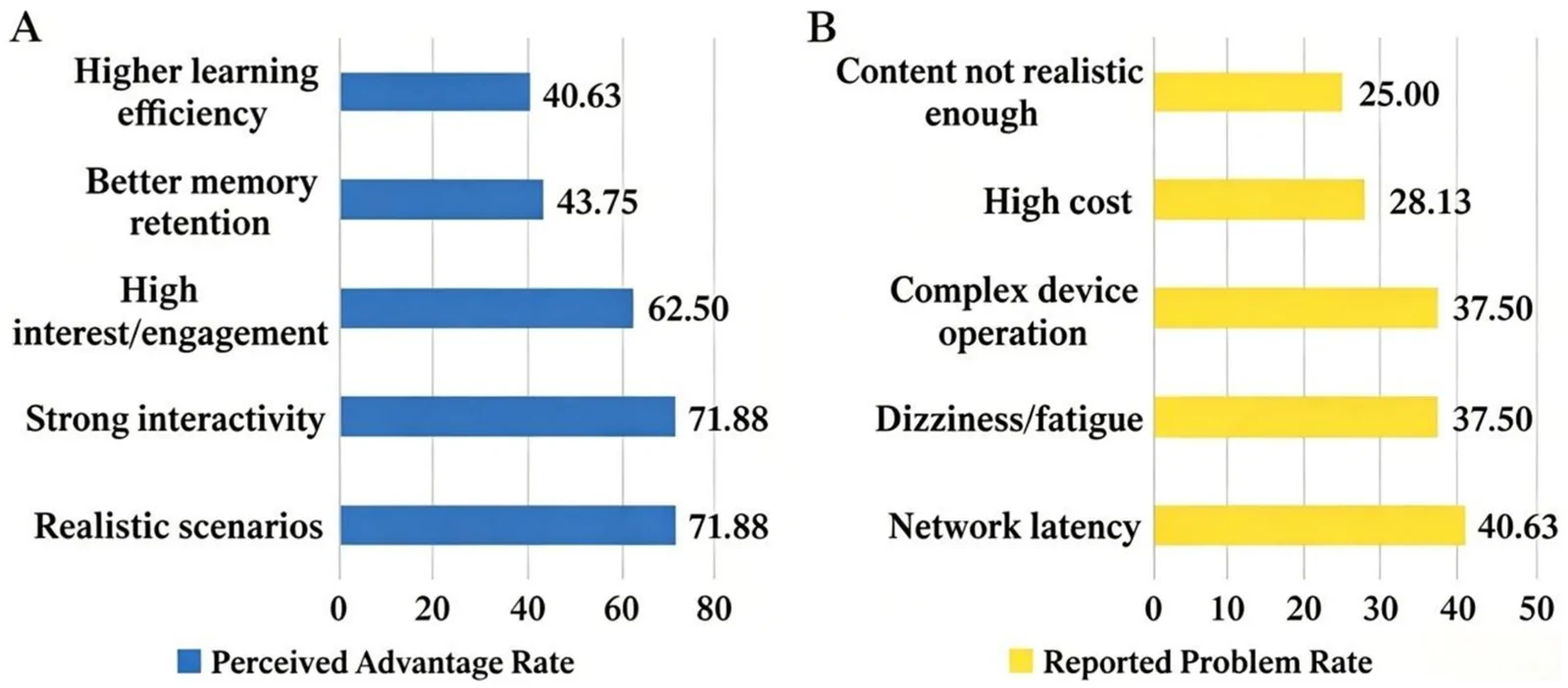
Perceived affordances and constraints of prior VR learning experiences among participants with VR exposure. (**A**) Perceived advantages of VR learning; (**B**) Reported constraints of VR learning.

**Figure 6:**
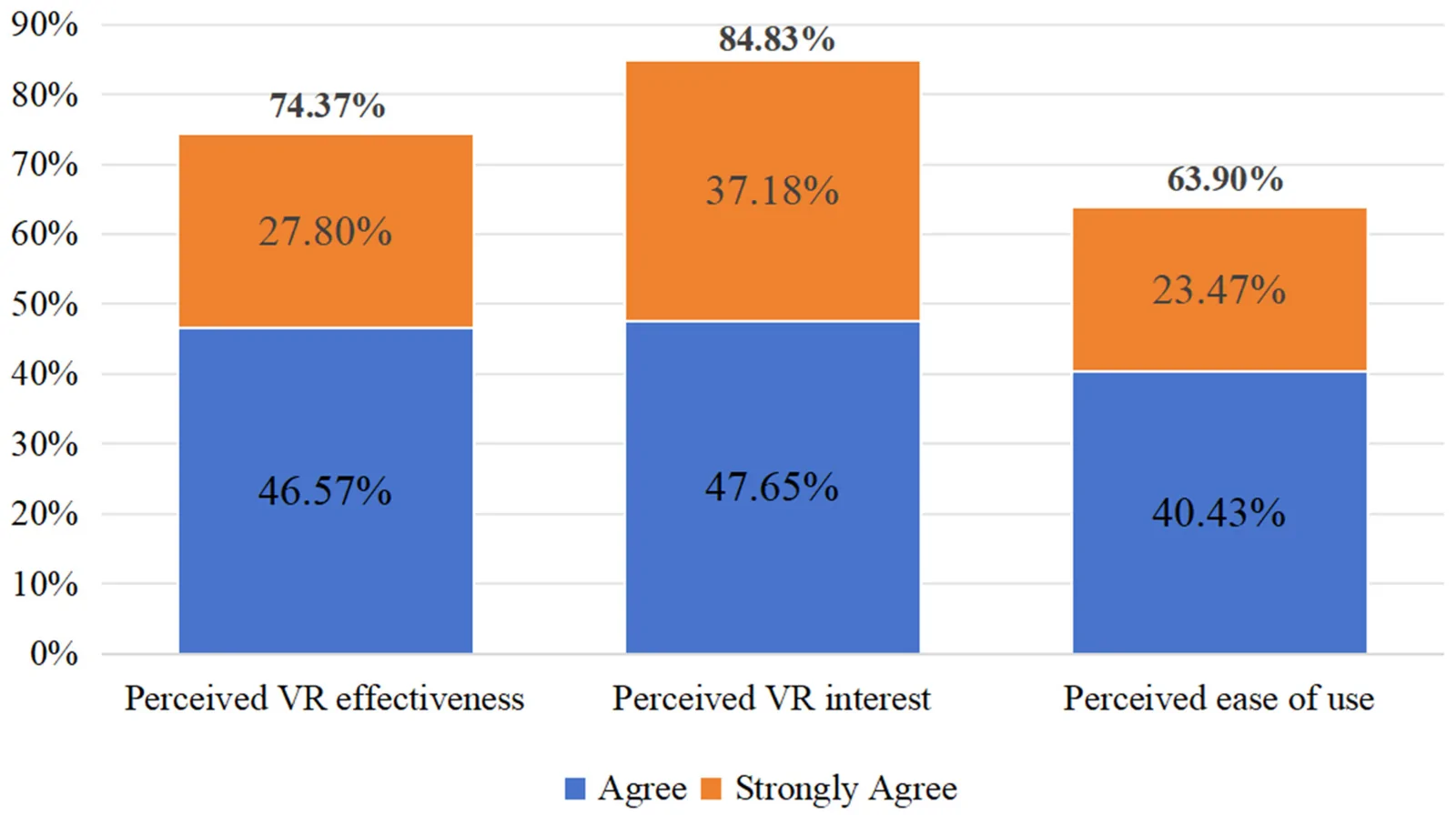
Students’ Recognition of VR Potential in Foreign Language Education.

**Figure 7:**
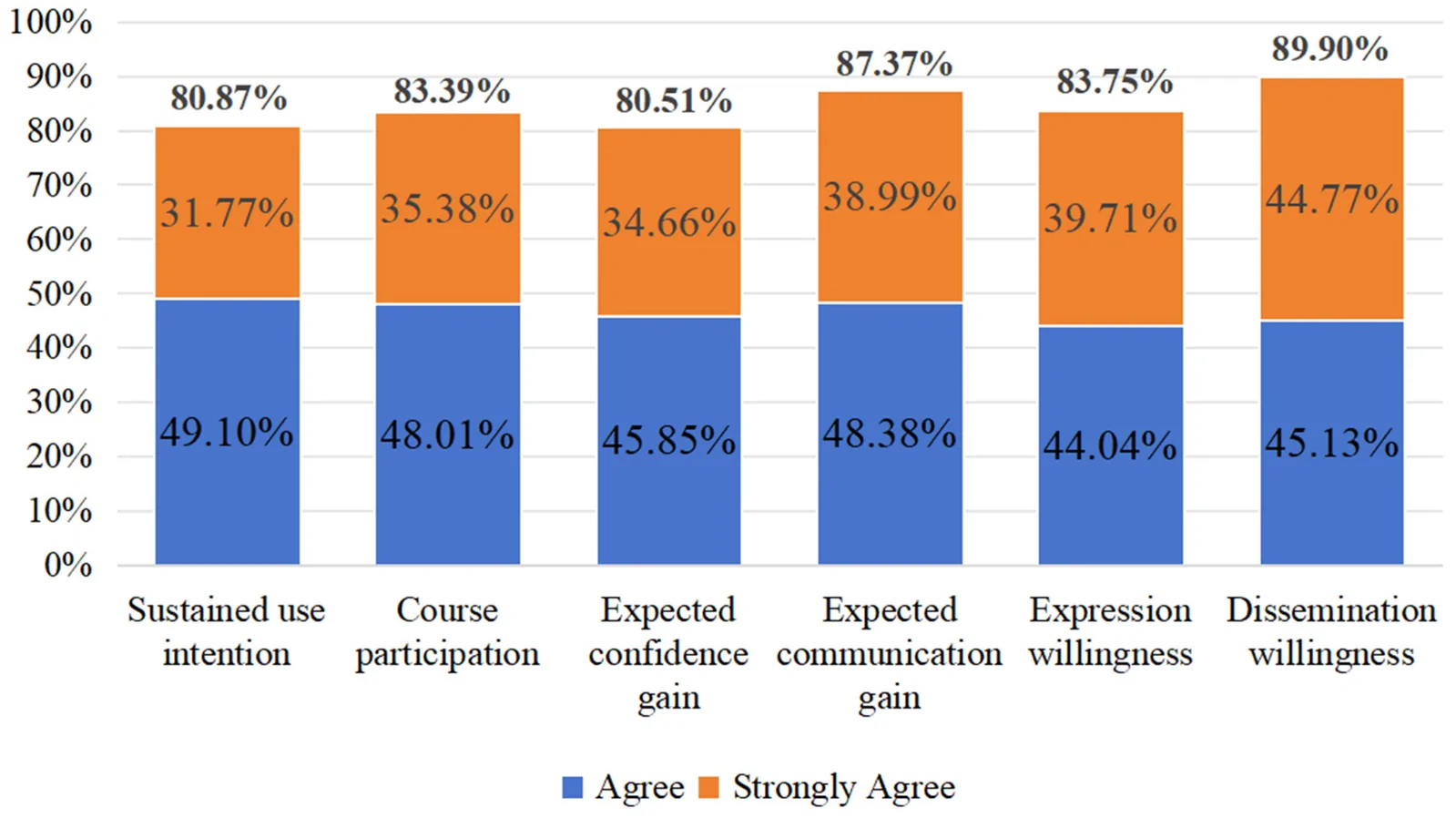
Students’ Acceptance and Expectations of Integrated VR System.

**Figure 8:**
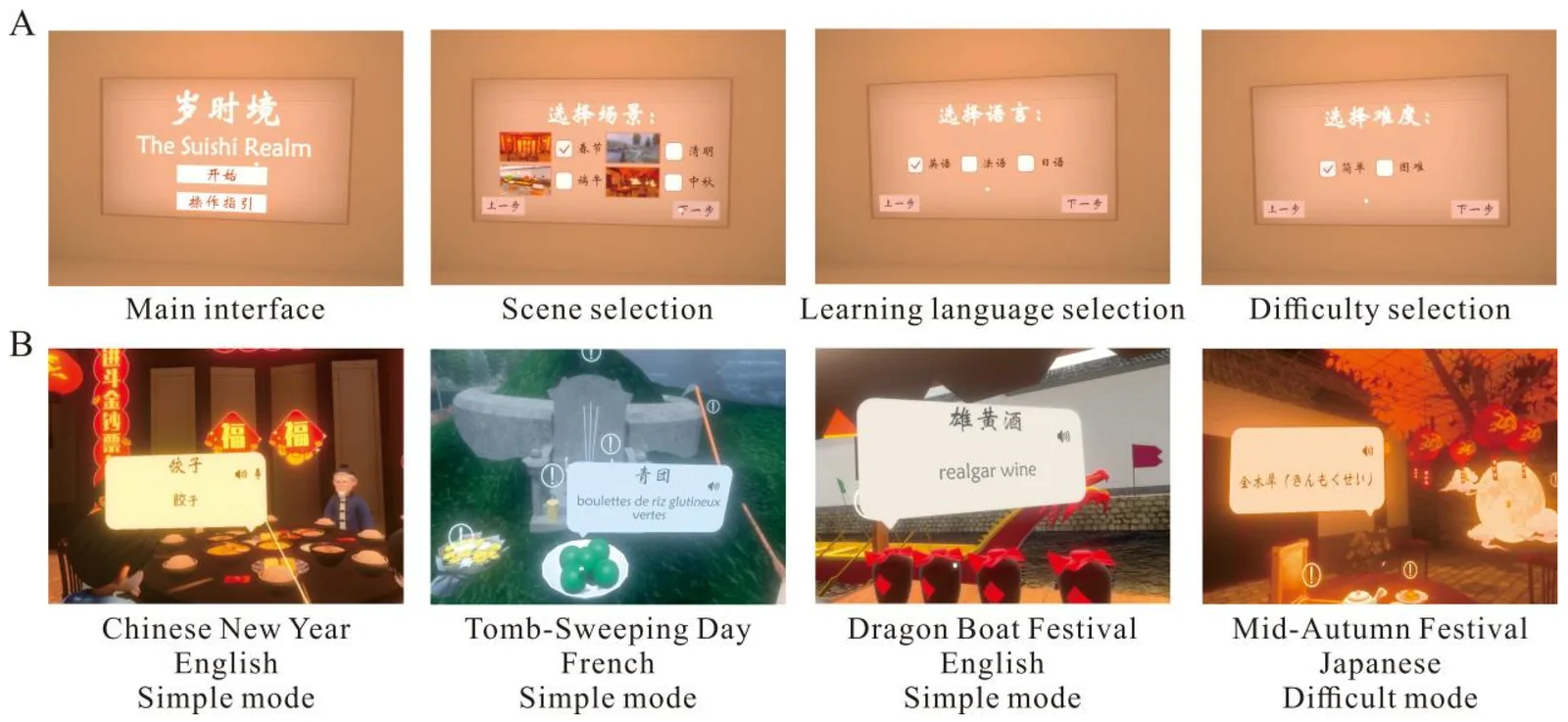
Overview of the VR-assisted foreign language learning system (The Suishi Realm) interface (**A**) and different cultural learning scenarios (**B**).

**Figure 9:**
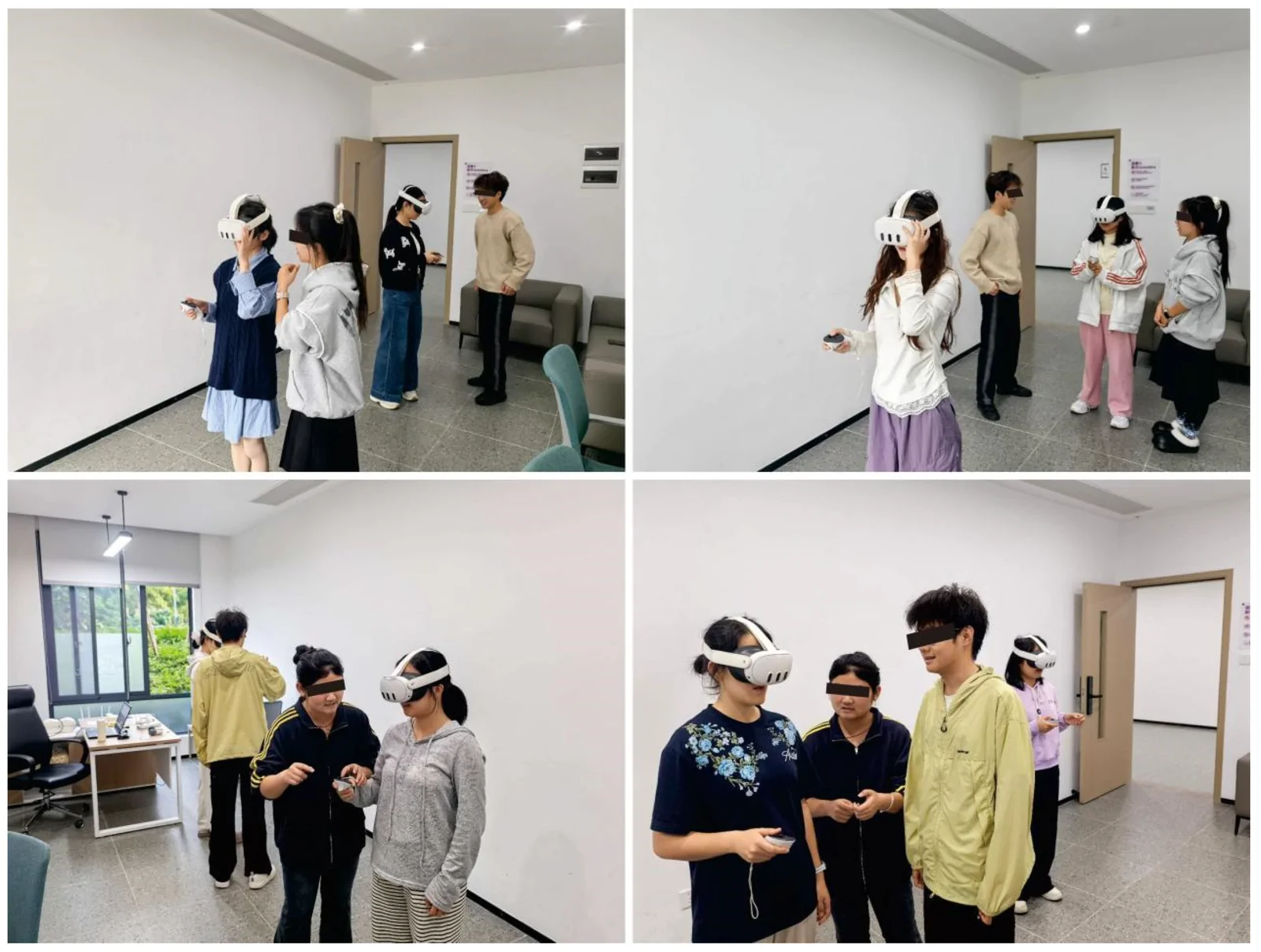
On-site implementation of the “The Suishi Realm” immersive VR-assisted language learning experience session.

**Table 1:** Item-Level Descriptive Statistics for the CETC-Integrated VR-Supported Foreign Language Learning Needs-Analysis Questionnaire in Study 1. All items were rated from 1 = strongly disagree to 5 = strongly agree. *N* = 277 for all items. Agree/strongly agree (%) refers to the percentage of participants selecting 4 or 5. CETC = Chinese Excellent Traditional Culture; VR = virtual reality.

Original Item No.	Content Domain	English Item Wording	*M* ± *SD*	Agree/Strongly Agree (%)
18	Culture-language relation	Foreign language learning is not only language learning, but also a process of understanding culture.	4.32 ± 0.81	91.34
19	Cultural comprehension	Understanding CETC can help me better understand related vocabulary in foreign-language contexts.	4.14 ± 0.93	81.22
20	CETC input	Foreign language textbooks contain relatively little content related to CETC.	3.67 ± 1.06	61.01
21	CETC learning need	I hope that foreign language courses will include more content introducing CETC.	4.03 ± 0.91	77.61
22	CETC learning interest	I am interested in foreign language learning that integrates CETC.	4.08 ± 0.90	78.34
23	Expression willingness	I am willing to introduce CETC in a foreign language.	4.19 ± 0.82	83.75
24	Cultural pride	Learning CETC in foreign language classes can enhance my sense of cultural pride.	4.25 ± 0.81	83.30
25	Expression difficulty	I am concerned that I may not be able to accurately express CETC in a foreign language.	4.01 ± 1.01	78.34
26	Dissemination willingness	I am willing to better disseminate CETC through foreign language learning.	4.31 ± 0.76	89.90
27	Perceived VR effectiveness	I think using VR to learn a foreign language can improve learning effectiveness.	3.99 ± 0.82	74.37
28	Perceived VR interest	I think VR learning may be more interesting than traditional classroom learning.	4.18 ± 0.80	84.83
29	Perceived ease of use	I can easily learn how to use a VR system.	3.79 ± 0.91	63.90
30	Sustained use intention	I am willing to use, over the long term, a foreign language learning support system that integrates CETC content with VR technology.	4.08 ± 0.81	80.87
31	Course participation	If my school provided such opportunities, I would actively participate in VR-based foreign language courses related to CETC.	4.14 ± 0.82	83.39
32	Immersive scenario need	I hope the VR system can provide virtual cultural scenarios, such as tea art, ancient architecture, and festival activities, so that I can experience the charm of Chinese culture more intuitively.	4.29 ± 0.75	89.17
33	Virtual interaction need	I hope VR learning will allow me to engage in foreign-language cultural communication with virtual characters.	4.25 ± 0.77	63.90
34	Speech interaction need	I hope the system can provide speech recognition to help correct foreign-language pronunciation and expression.	4.32 ± 0.73	91.70
35	Adaptive difficulty need	I hope the system can adjust the learning difficulty according to my proficiency level.	4.40 ± 0.73	91.33
36	Personalized feedback need	I hope the system can generate learning reports or personalized feedback.	4.39 ± 0.69	93.50
37	Intercultural understanding	I can understand differences in people’s ways of expression across different cultural backgrounds.	4.18 ± 0.79	79.06
38	Communication difficulty in conflict	When encountering cultural conflict, I am unable to communicate and explain appropriately using suitable language.	3.70 ± 1.03	64.26
39	Practice opportunity need	I think I need more opportunities to practice expressing CETC-related content.	4.18 ± 0.79	84.47
40	Expected confidence gain	I think learning a foreign language in a VR environment can improve my confidence in cross-cultural communication.	4.10 ± 0.85	80.51
41	Expected communication gain	I hope to improve my cross-cultural communication ability by practicing in VR scenarios in the future.	4.24 ± 0.75	87.37

**Table 2:** Alignment across Study 1 findings, Study 2 design features, and Study 3 learner-perceived support.

Study 1 Challenge/Need	Study 2 Design Feature	Study 3 Learner-Perceived Support
Insufficient contextualized CETC input	Immersive festival scenarios and culturally meaningful objects	Learners perceived the scenes as making cultural content more concrete and memorable
Difficulty mapping cultural meanings to linguistic forms	Object-based vocabulary learning and structured dialogue practice	Learners perceived that words, objects, and dialogue prompts helped connect cultural meanings with language expression
Affective barriers, such as nervousness and low confidence	Scaffolded mode, repeatable practice, pronunciation playback, formative AI scoring	Learners perceived the VR environment as a relatively low-risk space for practice
Need for more cross-cultural communication practice	Virtual foreign peer, scenario-based interaction, target-language dialogue tasks	Learners wanted richer NPC dialogue and more daily-life communication scenarios
Need for personalized support and feedback	Step-by-step prompts, replay function, AI-based oral scoring, performance summary	Learners valued immediate support but suggested more sentence-level scaffolding and oral feedback

## Data Availability

The data presented in this study are available on request from the corresponding author. The data are not publicly available due to ethical and privacy restrictions, as they include participants’ questionnaire responses and interview recordings containing potentially identifiable personal information. Access to the data is therefore restricted to protect participant confidentiality.

## Supplementary Materials

The following supporting information can be downloaded at: https://www.mdpi.com/article/10.3390/bs16101718/s1.
