# Supplementary material for: A Design-Based VR-Assisted Learning System for Culturally Grounded Meaning Construction in Foreign Language Learning: Integrating Chinese Excellent Traditional Culture
Source: Behav Sci (Basel). 2026 Sep 22;16(10):1718. doi: 10.3390/bs16101718 (PMC13701391; doi:10.3390/bs16101718)
Supplement: Supplementary file 1 [file behavsci-16-01718-s001.zip › File S1.pdf]

中华优秀传统文化融入外语学习现状调查

亲爱的同学：

您好！

感谢您愿意抽出宝贵时间参与本次调查，本问卷旨在了解大学生在外语学习过程中对中华优秀传统文化融入教学的认知、态度与体验，以及对虚拟现实（VR）技术应用于外语学习的接受程度与功能需求。

本研究仅用于学术研究，所有信息将严格保密，不涉及个人隐私。作答时间约3分钟，请您根据真实体验填写。感谢您的参与！

1.您的性别： [单选题] *

○男　　○女

2.您来自： [单选题] *

○城镇

○农村

3.您的年龄段： [单选题] *

○18岁以下　　○18~25　　○26~30　　○30岁以上

4.您的年级： [单选题] *

○大一

○大二

○大三

○大四

○研究生

○博士

○其他 ________________ *

5.您的专业类型： [多选题] *

☐ 语言类（英语、法语、日语等） ☐ 人文社科类 ☐ 理工类

☐ 艺体类 ☐ 经管类 ☐ 其他 _____*

6.您系统学习的过的外语有： [多选题] *

☐英语 ☐法语 ☐日语 ☐其他 _____*

7.您的外语学习年限： [多选题] *

（若第 6 题选择了两个或两个以上的选项，则此题在选项后备注对应的语言。）

☐3 年及以下 ☐4~6 年 ☐7~10 年 ☐10 年以上

_____________ _____________ _____________ _____________

8.您的外语水平： [单选题] *

○ 通过 CET-4 ○ 通过 CET-6 ○ 通过 TEM-4/TEM-8

○ 其他国际认证（如 DELF、JLPT 等） ○ 暂未通过考试

9.您是否接触过 VR（虚拟现实）技术？ [单选题] *

○是 (请跳至第 10 题) ○否 (请跳至第 17 题)

10.您使用 VR 的频率： [单选题] *

○ 经常使用 ○ 偶尔使用 ○ 仅短暂体验过

11.您使用 VR 的主要场景： [单选题] *

○ 游戏娱乐 ○ 学习培训 ○ 旅游体验 ○ 社交平台 ○ 其他 ____

12.您在使用 VR 时的整体体验感受： [单选题] *

○ 非常沉浸 ○ 较为沉浸 ○ 一般 ○ 略显不适 ○ 明显不适

13.您是否在学习或教学活动中使用过 VR？ [单选题] *

○ 是 (请跳至第 14 题)　　○ 否 (请跳至第 17 题)

14.请描述您使用 VR 学习的内容或课程： [填空题] *

____

15.使用 VR 学习时，您是否出现以下问题（可多选）： [多选题] *

☐ 头晕/疲劳　☐ 设备操作复杂　☐ 内容不够真实

☐ 网络延迟　☐ 成本高　☐ 其他 __________

16.您认为 VR 学习最大的优点是（可多选）： [多选题] *

☐ 场景逼真　☐ 互动性强　☐ 趣味性高

☐ 记忆效果好　☐ 学习效率高　☐ 其他 ___________

17.您是否愿意使用 VR 辅助外语学习？ [单选题] *

○ 非常愿意　○ 较愿意　○ 一般　○ 不太愿意　○ 完全不愿意

请根据您的真实想法选择最符合的选项：[矩阵单选题] *

	非常不同意	不同意	一般	同意	非常同意
18.外语学习不仅是语言学习，更是理解文化的过程。	○	○	○	○	○
19. 了解中华优秀传统文化能帮助我更好地理解外语语境中的相关词汇。	○	○	○	○	○
20. 外语教材中与中华优秀传统文化相关内容较少。	○	○	○	○	○

21. 我希望外语课程中增加介绍中华优秀传统文化的内容。	○	○	○	○	○
22. 我对融合了中华优秀传统文化的外语学习有兴趣。	○	○	○	○	○

请根据您的真实想法选择最符合的选项：[矩阵单选题] *

	非常不同意	不同意	一般	同意	非常同意
23.我愿意用外语介绍中华优秀传统文化。	○	○	○	○	○
24.在外语课堂中学习中华优秀传统文化能让我增强文化自豪感。	○	○	○	○	○
25.我担心自己无法用外语准确表达中华优秀传统文化。	○	○	○	○	○
26.我愿意通过学习外语更好地传播中华优秀传统文化。	○	○	○	○	○

请根据您的真实想法选择最符合的选项：[矩阵单选题] *

	非常不同意	不同意	一般	同意	非常同意
27.我认为使用 VR 学习外	○	○	○	○	○

语能提高学习效果。					
28.我认为 VR 学习可能比传统课堂更有趣。	○	○	○	○	○
29.我能容易地学习如何使用 VR 系统。	○	○	○	○	○
30.我愿意在外语学习中长期使用将中华优秀传统文化内容与 VR 技术结合的外语学习辅助系统。	○	○	○	○	○
31.如果学校能提供相关机会，我会积极参与和中华优秀传统文化相关的 VR 外语课程。	○	○	○	○	○

请根据您的真实想法选择最符合的选项：[矩阵单选题] *

	非常不同意	不同意	一般	同意	非常同意
32.我希望 VR 系统能提供虚拟文化场景（如茶艺、古建筑、节日活动），让我更直观感受中国文化的魅力。	○	○	○	○	○
33.我希望 VR 学习能让我与虚拟人物进行外语文化交流。	○	○	○	○	○

34.我希望系统提供语音识别功能，帮助纠正外语发音与表达。	○	○	○	○	○
35.我希望系统能根据我的水平调整学习难度。	○	○	○	○	○
36.我希望系统能生成学习报告或个性化反馈。	○	○	○	○	○

请根据您的真实想法选择最符合的选项：[矩阵单选题] *

	非常不同意	不同意	一般	同意	非常同意
37.我能理解不同文化背景下人们表达方式的差异。	○	○	○	○	○
38.当遇到文化冲突时，我无法用合适的语言方式进行沟通和解释。	○	○	○	○	○
39.我认为自己需要有更多的机会练习表达中华优秀传统文化内容。	○	○	○	○	○
40.我认为在 VR 环境下学习外语可以提升我在跨文化交流中的表达信心。	○	○	○	○	○
41.我希望未来能通过 VR 场景练习提升跨文化沟通能力。	○	○	○	○	○

融入中华优秀传统文化的 VR 辅助语言学习系统构建

访谈提纲

同学你好，非常感谢你参与本次访谈。该访谈是我们正在开展的一项研究中的一部分，本研究旨在构建并验证一套融入中华优秀传统文化的 VR 辅助大学生第二外语学习系统，以提升外语学习效果、增强文化自信，并促进学生在跨文化情境中准确表达中华优秀传统文化的能力。鉴于现有外语学习场景中存在文化内容融入不足、学习动机和参与度有限，以及缺乏沉浸式跨文化体验等问题，本研究希望通过 VR 技术提供真实、沉浸、互动的学习环境，从而推动外语学习与中华文化传播的深度融合。下面我们将根据你刚才参与的 VR 学习体验，与你进行一次访谈。所有访谈数据均会匿名保存、访谈内容也将完全保密。

请简单介绍一下你的专业、年级、主要外语类型，以及你是否具有 VR 使用经验。

下面的访谈将围绕你的外语学习经历、对中华文化的态度，以及使用 VR 学习系统的体验展开，你可以根据真实想法自由表达。

1. 在你的外语学习过程中，中华传统文化内容通常以什么形式出现？（教材/课堂/短视频/影视/几乎没有）

2. 你觉得这些文化内容是否促进外语学习动机？

3. 在用外语介绍中国文化时，你的感受通常是自信、紧张、尴尬还是其他？为什么？

4. 是什么因素影响你使用外语介绍中国文化的意愿？（如知识、词汇、自信、场合等）

5. 你是否曾经成功或失败地向外国朋友介绍中国文化？能分享经历吗？如果没有相关经历，你是否愿意向外国朋友介绍中华优秀传统文化？

6. 在跨文化交流情境（真实/模拟）中，你通常会面临哪些沟通障碍？

7. 你认为自己目前的跨文化理解能力和表达能力处于什么水平？

8. 在文化误解或冲突出现时，你通常会怎么处理？

9. 你希望未来学习中有哪些跨文化沟通训练方式？（如角色扮演、模拟对话、情境演练等）

---

10. 你对整个 VR 学习体验的第一印象是什么？

11. 如果让你用一句话描述 VR 学习体验，你会怎么说？

12. 在沉浸感方面，你体验到哪些具体的“真实感”或“代入感”？

13. 在使用过程中，是否出现不适（眩晕、头晕、疲劳）？程度如何？

14. 相较于传统教学，VR 辅助语言学习系统是否帮助你建立文化背景知识，从而更容易进行跨文化解释？

15. 你觉得开展长期的 VR 辅助语言学习能否提升你在真实跨文化环境中的沟通能力？为什么？

16. 哪种跨文化场景是你最希望 VR 增加的？（如餐桌礼仪、节庆交流、学术沟通等）

17. 对于语言学习部分，你需要更多：单词/短语解释？句型示例？口语反馈？文化背景讲解？

18. 该 VR 系统中你最喜欢和最不喜欢的设计分别是什么？

19. 如果让你设计下一版系统，你会重点改进哪些方面？

Questionnaire on the Integration of Chinese Excellent Traditional Culture into Foreign Language Learning

Dear student,
Hello! Thank you for taking the time to participate in this survey. This questionnaire aims to investigate college students' perceptions, attitudes and experiences regarding the integration of Chinese Excellent Traditional Culture (CETC) into foreign language teaching, as well as their acceptance and functional demands for the application of virtual reality (VR) technology in foreign language learning.

This study is for academic research only. All information will be kept strictly confidential and will not involve personal privacy. The survey takes approximately 3 minutes to complete. Please fill in the form according to your real experience. Thank you for your participation!

Scoring Instructions

The 5-point Likert scale items are scored as follows: 1 = Strongly disagree, 2 = Disagree, 3 = Neutral, 4 = Agree, 5 = Strongly agree. A higher score indicates a stronger level of agreement with the corresponding statement.

Demographic, single-choice and multiple-choice items are analyzed by frequency and percentage.

1. Your gender: [Single choice]

○ Male
○ Female

2. Your place of origin: [Single choice]

○ Urban area
○ Rural area

3. Your age group: [Single choice]

○ Under 18 years old
○ 18–25 years old
○ 26–30 years old
○ Over 30 years old

4. Your academic year: [Single choice]

○ Freshman
○ Sophomore

○ Junior

○ Senior

○ Postgraduate student

○ Doctoral student

○ Other

5. Your disciplinary background: [Multiple choice]

□ Language-related majors (English, French, Japanese, etc.)

□ Humanities and Social Sciences

□ Science, Technology, Engineering and Mathematics (STEM)

□ Arts and Physical Education

□ Economics and Management

□ Other

6. Foreign languages you have learned systematically: [Multiple choice]

□ English

□ French

□ Japanese

□ Other

7. Years of foreign language learning: [Multiple choice]

(If you selected two or more options in Question 6, please note the corresponding language after each option.)

□ 3 years or less

□ 4–6 years

□ 7–10 years

□ More than 10 years

8. Your foreign language proficiency: [Single choice]

○ Passed CET-4

○ Passed CET-6

○ Passed TEM-4 / TEM-8

○ Other international certification (e.g., DELF, JLPT, etc.)

○ Have not passed any proficiency test yet

9. Have you ever been exposed to virtual reality (VR) technology? [Single choice]

○ Yes (Please skip to Question 10)

○ No (Please skip to Question 17)

10. How often do you use VR? [Single choice]

○ Frequently

○ Occasionally

○ Only brief experience

11. Main scenarios of your VR use: [Single choice]

○ Gaming and entertainment

○ Learning and training

○ Tourism experience

○ Social platform

○ Other

12. Your overall experience when using VR: [Single choice]

○ Highly immersive

○ Relatively immersive

○ Average

○ Slightly uncomfortable

○ Obviously uncomfortable

13. Have you ever used VR in learning or teaching activities? [Single choice]

○ Yes (Please skip to Question 14)

○ No (Please skip to Question 17)

14. Please describe the content or courses you learned using VR: [Fill in the blank]

15. Have you encountered the following problems when learning with VR? [Multiple choice]

□ Dizziness / fatigue

□ Complex device operation

□ Unrealistic content

□ Network latency

□ High cost

□ Other

16. What do you think are the biggest advantages of VR-based learning? [Multiple choice]

□ Realistic scenarios

□ Strong interactivity

□ High level of interest

□ Good memory retention

□ High learning efficiency

□ Other

17. Are you willing to use VR to assist foreign language learning? [Single choice]

○ Very willing
○ Relatively willing
○ Neutral
○ Reluctant
○ Completely unwilling

Please select the option that best matches your true opinion: [Matrix single choice]

	Strongly disagree	Disagree	Neutral	Agree	Strongly agree
18. Foreign language learning is not only language learning, but also a process of understanding culture.	○	○	○	○	○
19. Understanding CETC can help me better understand related vocabulary in foreign-language contexts.	○	○	○	○	○
20. Foreign language	○	○	○	○	○

textbooks contain relatively little content related to CETC.					
21. I hope that foreign language courses will include more content introducing CETC.	○	○	○	○	○
22. I am interested in foreign language learning that integrates CETC.	○	○	○	○	○
23. I am willing to introduce CETC in a foreign language.	○	○	○	○	○
24. Learning CETC in foreign language	○	○	○	○	○

classes can enhance my sense of cultural pride.					
25. I am concerned that I may not be able to accurately express CETC in a foreign language.	○	○	○	○	○
26. I am willing to better disseminate CETC through foreign language learning.	○	○	○	○	○
27. I think using VR to learn a foreign language can improve learning effectivenes s.	○	○	○	○	○

28. I think VR learning may be more interesting than traditional classroom learning.	○	○	○	○	○
29. I can easily learn how to use a VR system.	○	○	○	○	○
30. I am willing to use, over the long term, a foreign language learning support system that integrates CETC content with VR technology.	○	○	○	○	○
31. If my school provided such opportunitie s, I would	○	○	○	○	○

actively participate in VR-based foreign language courses related to CETC.					
32. I hope the VR system can provide virtual cultural scenarios, such as tea art, ancient architecture, and festival activities, so that I can experience the charm of Chinese culture more intuitively.	○	○	○	○	○
33. I hope VR learning will allow me to engage in foreign-language cultural	○	○	○	○	○

communication with virtual characters.					
34. I hope the system can provide speech recognition to help correct foreign-language pronunciation and expression.	○	○	○	○	○
35. I hope the system can adjust the learning difficulty according to my proficiency level.	○	○	○	○	○
36. I hope the system can generate learning reports or personalized feedback.	○	○	○	○	○

37. I can understand differences in people's ways of expression across different cultural backgrounds.	○	○	○	○	○
38. When encountering cultural conflict, I am unable to communicate and explain appropriately using suitable language.	○	○	○	○	○
39. I think I need more opportunities to practice expressing CETC-related content.	○	○	○	○	○
40. I think learning a	○	○	○	○	○

foreign language in a VR environment can improve my confidence in cross-cultural communication.					
41. I hope to improve my cross-cultural communication ability by practicing in VR scenarios in the future.	○	○	○	○	○

Interview Guide for a CETC-Integrated VR-Assisted Language Learning System

Hello, thank you very much for participating in this interview. This interview is part of an ongoing research study. This study aims to develop and validate a VR-assisted second foreign language learning system integrated with Chinese Excellent Traditional Culture for college students, to improve foreign language learning outcomes, enhance cultural confidence, and promote students' ability to accurately express Chinese Excellent Traditional Culture in cross-cultural contexts.

Given the problems of insufficient cultural content integration, limited learning motivation

and engagement, and lack of immersive cross-cultural experience in existing foreign language learning scenarios, this study intends to provide an authentic, immersive and interactive learning environment through VR technology, so as to promote the deep integration of foreign language learning and Chinese cultural communication. Next, we will conduct an interview with you based on the VR learning experience you just participated in. All interview data will be stored anonymously, and the interview content will be kept completely confidential.

Please briefly introduce your major, academic year, main foreign language, and whether you have prior experience using VR.

The following interview will focus on your foreign language learning experience, your attitude toward Chinese culture, and your experience using the VR learning system. You may express yourself freely according to your true thoughts.

1. In your foreign language learning process, in what forms does Chinese traditional culture usually appear? (Textbooks / classroom instruction / short videos / films and television / almost none)

2. Do you think such cultural content promotes your motivation for foreign language learning?

3. When introducing Chinese culture in a foreign language, do you usually feel confident, nervous, embarrassed, or something else? Why?

4. What factors influence your willingness to introduce Chinese culture in a foreign language? (e.g., knowledge, vocabulary, confidence, situation, etc.)

5. Have you ever had a successful or unsuccessful experience introducing Chinese culture to foreign friends? Can you share the experience? If you have no relevant experience, are you willing to introduce Chinese Excellent Traditional Culture to foreign friends?

6. In cross-cultural communication situations (real or simulated), what communication barriers do you usually face?

7. What level do you think your current cross-cultural understanding ability and expression ability are at?

8. When cultural misunderstanding or conflict occurs, how do you usually handle it?

9. What forms of cross-cultural communication training do you hope to have in future learning? (e.g., role play, simulated dialogue, scenario practice, etc.)

10. What is your first impression of the overall VR learning experience?

11. If you had to describe the VR learning experience in one sentence, what would you say?

12. In terms of immersion, what specific feelings of "realism" or "sense of presence" did you experience in VR?

13. Did you experience any discomfort during use (vertigo, dizziness, fatigue)? To what extent?

14. Compared with traditional teaching, does the VR-assisted language learning system help you build cultural background knowledge, so that it is easier to carry out cross-cultural interpretation?

15. Do you think long-term VR-assisted language learning can improve your communication ability in real cross-cultural environments? Why?

16. What kind of cross-cultural scenario do you most want the VR system to add? (e.g., table manners, festival interaction, academic communication, etc.)

17. For the language learning part, what do you need more of: word / phrase explanation? example sentences? oral feedback? cultural background explanation?

18. What are your most liked and least liked design features of this VR system respectively?

19. If you were to design the next version of the system, what aspects would you focus on improving?
