# Supplementary material for: A Design-Based VR-Assisted Learning System for Culturally Grounded Meaning Construction in Foreign Language Learning: Integrating Chinese Excellent Traditional Culture
Source: Behav Sci (Basel). 2026 Sep 22;16(10):1718. doi: 10.3390/bs16101718 (PMC13701391; doi:10.3390/bs16101718)
Supplement: Supplementary file 1 [file behavsci-16-01718-s001.zip › File S2.pdf]

File S2

Table S1 Mapping Between Interview Questions and Analytic Modules

Interview question	Main focus	Analytic module	Analytic function
Q1	Forms in which Chinese traditional culture appeared in prior foreign language learning, such as textbooks, classroom instruction, short videos, films, or little exposure	Module 1: Prior exposure to CETC in foreign language learning	Identifies learners' baseline exposure to culturally related language input
Q2	Whether such cultural content promoted motivation for foreign language learning	Module 1: Prior exposure to CETC in foreign language learning	Examines whether CETC functioned as a motivational resource
Q3	Learners' feelings when introducing Chinese culture in a foreign language, such as confidence, nervousness, embarrassment, or other emotions	Module 2: Cultural confidence and foreign-language expression	Identifies affective responses during culturally grounded language expression
Q4	Factors influencing willingness to introduce Chinese culture in a foreign language, including knowledge, vocabulary, confidence, and communicative context	Module 2: Cultural confidence and foreign-language expression	Identifies cognitive, linguistic, affective, and situational factors shaping expression
Q5	Previous successful or unsuccessful experiences of introducing Chinese culture to foreigners, or willingness to do so in the future	Module 2: Cultural confidence and foreign-language expression	Examines prior communicative practice and willingness to express CETC
Q6	Communication barriers in real or simulated cross-cultural contexts	Module 3: Cross-cultural communication barriers and training needs	Identifies perceived barriers in cross-cultural communication
Q7	Self-perceived level of cross-cultural understanding	Module 3: Cross-cultural	Compares learners' receptive

	and expression ability	communication barriers and training needs	understanding and productive expression ability
Q8	Strategies for dealing with cultural misunderstanding or conflict	Module 3: Cross-cultural communication barriers and training needs	Examines learners' intercultural coping strategies and awareness of cultural difference
Q9	Preferred forms of cross-cultural communication training, such as role play, simulated dialogue, or scenario-based practice	Module 3: Cross-cultural communication barriers and training needs	Identifies learners' preferred pedagogical supports for intercultural communication
Q10	First impression of the VR learning experience	Module 4: VR learning experience and system refinement needs	Captures learners' overall initial evaluation of the VR system
Q11	One-sentence description of the VR learning experience	Module 4: VR learning experience and system refinement needs	Captures learners' general experiential judgment of VR-supported learning
Q12	Specific sense of realism, immersion, or presence experienced in VR	Module 4: VR learning experience and system refinement needs	Examines perceived immersive affordances of the VR environment
Q13	Physical discomfort during use, such as dizziness, fatigue, or nausea	Module 4: VR learning experience and system refinement needs	Identifies usability, comfort, and technical limitations
Q14	Whether VR helped learners build cultural background knowledge and conduct cross-cultural explanation more easily	Module 4: VR learning experience and system refinement needs	Examines perceived support for contextualized cultural understanding and culturally grounded meaning construction
Q15	Whether long-term VR-assisted language learning could improve real cross-cultural communication	Module 4: VR learning experience and system	Examines perceived transfer potential from VR practice to real intercultural

	ability	refinement needs	communication
Q16	Desired additional cross-cultural scenarios, such as table manners, festival interaction, academic communication, or daily-life communication	Module 4: VR learning experience and system refinement needs	Identifies future scenario-design needs
Q17	Desired language-learning support, including vocabulary or phrase explanation, sentence examples, oral feedback, and cultural background explanation	Module 4: VR learning experience and system refinement needs	Identifies instructional scaffolding needs within the VR system
Q18	Most liked and least liked design features of the VR system	Module 4: VR learning experience and system refinement needs	Identifies perceived strengths and weaknesses of the current system
Q19	Suggestions for the next version of the system	Module 4: VR learning experience and system refinement needs	Identifies system refinement directions and learner-centered design expectations

Table S2 Framework-Guided Descriptive Qualitative Analysis Matrix

Analytic module	Corresponding questions	Evidence type	Main pattern	Representative quotation
Module 1: Prior exposure to CETC in foreign language learning	Q1–Q2	Confirmatory evidence	CETC appeared mainly in textbooks, classroom explanations, examination materials, short videos, films, or occasional extracurricular exposure, but it was often fragmented or supplementary.	“The textbook did not really include intangible cultural heritage, and in class we only watched some English expressions about Spring Festival or Lantern Festival customs.” (P1)
Module 1	Q1–Q2	Confirmatory evidence	Existing cultural input was sometimes exam-oriented, limited to isolated topics, or not organized into	“It usually appeared in reading passages, exam materials, or short videos, rather than as a complete

			sustained learning contexts.	learning context.” (P4)
Module 1	Q1–Q2	Explanatory evidence	CETC content increased motivation because it was familiar, meaningful, and linked to learners’ cultural identity.	“Learning English through content I am interested in makes English feel closer to life and motivates me to continue learning.” (P4)
Module 1	Q1–Q2	Improvement-oriented evidence	Learners expected richer and more systematic integration of Chinese cultural content rather than limited or stereotypical exposure.	“Some cultural content appears, but it is often strange, such as martial arts, and there is not much about festivals.” (P9)
Module 2: Cultural confidence and foreign-language expression	Q3–Q5	Confirmatory evidence	Learners were often confident in Chinese culture itself but lacked confidence in expressing culturally specific meanings accurately in a foreign language.	“I know a lot about Chinese traditional culture, but I usually cannot express it in a foreign language.” (P1)
Module 2	Q3–Q5	Confirmatory evidence	Vocabulary, specialized terminology, sentence formulation, and oral fluency were major barriers to cultural expression.	“I am very confident in the culture, but I become nervous because my vocabulary is not enough.” (P5)
Module 2	Q3–Q5	Confirmatory evidence	Some learners experienced a gap between intended cultural meaning and available foreign-language expression.	“I feel that the words I use cannot express what I really want to say.” (P7)
Module 2	Q3–Q5	Explanatory evidence	Learners valued contextualized support because it helped connect cultural objects, meanings, and foreign-language expressions.	“VR turns the words into concrete objects, and that makes them more memorable.” (P4)

Module 2	Q3–Q5	Improvement-oriented evidence	Learners wanted more sentence-level support, vocabulary explanations, and oral feedback.	“It would be better to add more sentences.” (P2)
Module 3: Cross-cultural communication barriers and training needs	Q6–Q9	Confirmatory evidence	Learners reported difficulties in listening comprehension, fast speech, unfamiliar accents, real-time response, and accurate expression.	“First, I may not understand what the other person says; even if I understand it, I may not have a good sentence pattern to respond.” (P2)
Module 3	Q6–Q9	Confirmatory evidence	Learners often perceived their understanding ability as stronger than their expressive ability.	“My cross-cultural expression ability is weak, while my understanding is relatively better.” (P1)
Module 3	Q6–Q9	Explanatory evidence	Role play, simulated dialogue, and scenario-based practice were perceived as useful because they could create more realistic and participatory communication contexts.	“Scenario-based dialogue would be the most effective.” (P3)
Module 3	Q6–Q9	Explanatory evidence	Learners expected communicative training that allowed them to practice in realistic or immersive contexts.	“It would be best to have a real kind of scene where I can use another language to learn and communicate.” (P5)
Module 3	Q6–Q9	Improvement-oriented evidence	Learners expected more daily-life communication, NPC interaction, AI-like correction, and dialogic practice.	“It could be like artificial intelligence and have simple conversations with me; if I make mistakes, it could correct me.” (P6)

Module 4: VR learning experience and system refinement needs	Q10–Q19	Confirmatory evidence	Learners perceived the VR system as novel, immersive, realistic, and more engaging than traditional instruction.	"The scene made me feel as if I were really there, and the fireworks in the Spring Festival scene were very clear." (P1)
Module 4	Q10–Q19	Confirmatory evidence	Most learners reported no major discomfort, but some noted headset weight, dizziness, or nausea.	"I felt a little dizzy and nauseous, so I ended the experience early." (P10)
Module 4	Q10–Q19	Explanatory evidence	VR supported contextualized understanding by connecting cultural objects, visual scenes, and language input.	"Compared with reading texts or watching videos, VR allows us to memorize and learn in a real scene." (P4)
Module 4	Q10–Q19	Explanatory evidence	Learners perceived VR as giving them more agency than traditional instruction.	"It gives the initiative to the learner; you can choose what you want to learn and listen to." (P9)
Module 4	Q10–Q19	Improvement-oriented evidence	The most common limitation was insufficient social interaction, especially the absence of people, NPCs, or dialogue.	"Some of the stalls had no people around them, which felt a little strange." (P1)
Module 4	Q10–Q19	Improvement-oriented evidence	Learners expected richer scenes, NPC communication, sentence-level scaffolding, oral feedback, and more realistic interaction.	"The most important improvement would be adding more NPCs for communication." (P2)

Table S3 Study 3 Themes and Illustrative Participant Quotations

**Study 3 theme**	**Illustrative quotation**
Fragmented prior exposure to CETC in	"The textbook did not really include intangible cultural heritage, and in class we only watched

foreign language learning	some English expressions about Spring Festival or Lantern Festival customs.” (P1)
Difficulty expressing familiar cultural knowledge in a foreign language	“I know a lot about Chinese culture, but I feel nervous because I cannot express it well in a foreign language.” (P1)
Gap between cultural meanings and linguistic expression	“I feel that the words I use cannot express what I really want to say.” (P7)
Perceived value of contextualized VR scenes	“VR turns the words into concrete objects, and that makes them more memorable.” (P4)
Perceived usefulness of immersive cultural experience	“It is more immersive than textbook learning; words and pictures together make it less rigid.” (P8)
Need for richer dialogue and social interaction	“It would be better to add more sentences.” (P2)
Need for NPC-based communication and feedback	“If there were NPCs for simple exchanges and corrections, it would be more helpful.” (P6)
Prototype limitations and comfort issues	“I felt dizzy and nauseous, so I ended the experience early.” (P10)
